# Transcriptional Response of Subcutaneous White Adipose Tissue to Acute Cold Exposure in Mice

**DOI:** 10.3390/ijms20163968

**Published:** 2019-08-15

**Authors:** Xiaojuan Liang, Jianfei Pan, Chunwei Cao, Lilan Zhang, Ying Zhao, Yiping Fan, Kui Li, Cong Tao, Yanfang Wang

**Affiliations:** 1 State Key Laboratory of Animal Nutrition, Institute of Animal Science, Chinese Academy of Agricultural Sciences, 100193 Beijing, China; 2 State Key Laboratory of Stem Cell and Reproductive Biology, Chinese Academy of Sciences, 100101 Beijing, China

**Keywords:** beige adipocyte, acute cold stimulation, transcriptional analysis, mice

## Abstract

Beige adipose tissue has been considered to have potential applications in combating obesity and its related metabolic diseases. However, the mechanisms of acute cold-stimulated beige formation still remain largely unknown. Here, transcriptional analysis of acute cold-stimulated (4 °C for 4 h) subcutaneous white adipose tissue (sWAT) was conducted to determine the molecular signatures that might be involved in beige formation. Histological analysis confirmed the appearance of beige adipocytes in acute cold-treated sWAT. The RNA-sequencing data revealed that 714 genes were differentially expressed (*p*-value < 0.05 and fold change > 2), in which 221 genes were upregulated and 493 genes were downregulated. Gene Ontology (GO) analyses showed that the upregulated genes were enriched in the GO terms related to lipid metabolic process, fatty acid metabolic process, lipid oxidation, fatty acid oxidation, etc. In contrast, downregulated genes were assigned the GO terms of regulation of immune response, regulation of response to stimulus, defense response, etc. The expressions of some browning candidate genes were validated in cold-treated sWAT and 3T3-L1 cell browning differentiation. In summary, our results illustrated the transcriptional response of sWAT to acute cold exposure and identified the genes, including *Acad11*, *Cyp2e1*, *Plin5*, and *Pdk2*, involved in beige adipocyte formation in mice.

## 1. Introduction

Obesity, a condition of excess fat accumulates in the body, and its related metabolic diseases, such as cardiovascular disease and diabetes, have become global health issues [1,2]. As an energy storage and endocrine organ, adipose tissue is critical for whole-body fat metabolism and energy homeostasis. Classically, mammals have two types of adipose tissues: brown adipose tissue (BAT) and white adipose tissue (WAT). BAT plays a critical role in adaptive thermogenesis and influences glucose and lipid metabolism due to its high mitochondrial content and high expression of uncoupling protein 1 (UCP1) [3]. In contrast, WAT, which contains relatively few mitochondria and expresses UCP1 at low levels, serves as a major reservoir for fuel storage [4]. In recent years, a new kind of adipocyte was identified in WAT upon various environmental cues, such as chronic cold stimulation, exercise, and treatment with rosiglitazone, the agonist of the major regulator of adipogenesis, peroxisome proliferator-activated receptor-γ (PPARγ) [3,4]. These adipocytes were named beige or “brite” (brown in white), and this cellular process was referred to as the “browning of WAT.” 

Beige adipocytes arise in WAT, and they present a mixture of structural and functional characteristics of both BAT and WAT [5]. Some experiments in rodents have provided strong evidence for the functions of beige adipocytes in improving thermogenesis, inducing resistance to obesity and attenuating components of metabolic syndrome. For example, Cohen et al. discovered that mice lacking functional beige adipocytes are more susceptible to obesity, insulin resistance, and hepatic steatosis when fed a high-fat diet (HFD) [6]. The transplantation of browning subcutaneous adipose tissue, which was induced by exercise, into mice fed a HFD restored glucose tolerance and insulin sensitivity [7]. As shown in the study by Harms et al., increased activity of beige or brown adipocytes in transgenic animal models resulted in resistance to obesity [8]. When the amount of BAT is substantially reduced, the induction of beige adipocytes in WAT restores the thermogenic response to cold and prevents the mice from a HFD induced obesity [9]. Several factors, such as exercise, irisin, and ectopic expression of *Prdm16*, have been reported to activate WAT browning, but cold exposure is a very efficient way of BAT activation and of WAT browning in mammals [10]. According to Piao et al., WAT browning was enhanced in BAT-deficient mice, and these mice exhibited significantly reduced adiposity [11]. Our previous study identified a number of novel miRNAs involved in the cold-induced sWAT browning in mice [12]. Collectively, these data demonstrate the critical role and potential applications of beige adipocytes in combating obesity and its related metabolic diseases. Therefore, explorations of the genetic basis of WAT browning have attracted increasing attention. 

In the present study, we induced beige adipocytes in sWAT by acute cold stimulation (4 °C for 4 h) in wild type C57BL/6J male mice. The transcriptional responses of sWAT were analyzed, and we found that genes associated with the GO terms of lipid metabolic process, lipid oxidation, PPAR signaling pathway and fatty acid transporter activity were significantly upregulated, and some genes were confirmed by quantitative real-time PCR (QPCR) analysis. Furthermore, the potential browning candidate genes were verified in 3T3-L1 differentiation toward beige adipocyte. 

## 2. Results

### 2.1. Acute Cold Exposure Stimulates sWAT Browning in Mice

**Figure 1:**
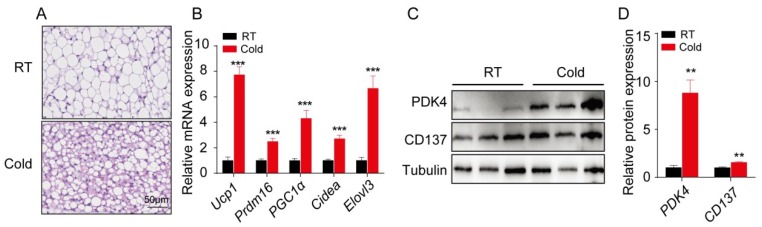
Acute cold exposure stimulates sWAT browning in mice. (**A**) Representative images of H&E staining in sections of sWAT from wild type control and cold-stimulated groups (*n* = 3). (**B**) The relative mRNA expression levels of brown/beige adipocyte marker genes (*Ucp1*, *Prdm16*, *Pgc1a*, *Cidea*, and *Elovl3*) in sWAT from control and cold treatment groups (*n* = 3). (**C**) Immunoblot analysis of PDK4 and CD137 in sWAT from both groups, one representative blot from 3 independent experiments is shown. (**D**) Quantitative data of immunoblot for PDK4 and CD137. Results represent the mean ±SEM, ** *p* < 0.01 and *** *p* < 0.001 for differences between control and cold treatment groups.

To explore whether acute cold stimulation (4 °C for 4 h) would induce formation of beige fat, H&E staining of sWAT from two groups of mice were conducted. Our data showed a smaller average adipocyte diameter in the cold-stimulated mice (Figure 1A). Next, we determined the changes in the expression of brown and beige adipocyte marker genes (*Ucp1*, *Prdm16*, *Pgc1a*, *Cidea*, and *Elovl3*). The RNA levels of these marker genes were significantly elevated in response to cold stimulation (Figure 1B). An increase of the beige adipocyte marker, CD137, and the brown adipocyte marker, PDK4, was observed in cold-treated sWAT at the protein level (Figure 1C); further confirming that sWAT browning occurred in cold-stimulated mice. The quantitative data from immunoblots are shown in Figure 1D. Collectively, our data showed that acute cold stimulation induced the formation of beige adipocytes in mouse sWAT.

### 2.2. Differentially Expressed Gene Screening and Gene Ontology Analysis in Cold-Treated sWAT

**Figure 2:**
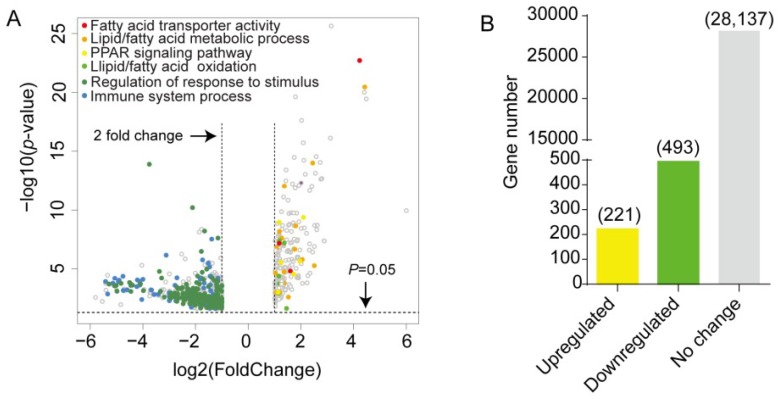

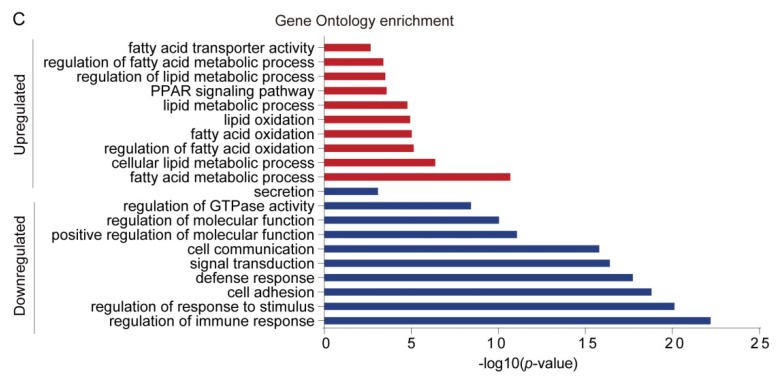
Acute cold exposure significantly alters the gene expression profiling of sWAT in mice. (**A**) Volcano plots show significance on the *y*-axis (-log 10, *p*-value) versus the gene expression ratio (log 2, fold change), and *p* = 0.05 is indicated by red dashed horizontal lines. The enriched GO terms were labeled with dots of different colors. (**B**) Histogram showing the number of differentially expressed genes (DEGs). (**C**) Gene Ontology analysis of DEGs. The *x*-axis represents −log10 (*p*-value).

To determine the transcriptional changes in sWAT in response to acute cold exposure, sWAT from control and cold-treated mice were collected and subjected to RNA-sequencing analyses. Differentially expressed genes (DEGs) were screened based on the following criteria; *p*-value < 0.05 and fold change > 2. Volcano plots show a broad overview of changes in gene expression between the RT and cold stimulation groups (Figure 2A). A total of 714 genes were observed to be significantly differentially expressed in sWAT from cold-stimulated mice. Two hundred twenty-one genes were upregulated and 493 genes were downregulated (Supplementary Tables S2 and S3 and Figure 2B). Furthermore, Gene Ontology (GO) analysis was performed with these DEGs, and the upregulated genes were enriched in the GO terms of lipid metabolic process, fatty acid metabolic process, lipid oxidation, fatty acid oxidation, and PPAR signaling pathway. In contrast, downregulated genes were assigned the GO terms of regulation of immune response, regulation of response to stimulus, cell adhesion, defense response, etc., as shown in Figure 2C.

### 2.3. Cold Exposure Significantly Alters the Expression of Genes Involved in the Fatty Acid Metabolic Process and Lipid Oxidation in sWAT

**Figure 3:**
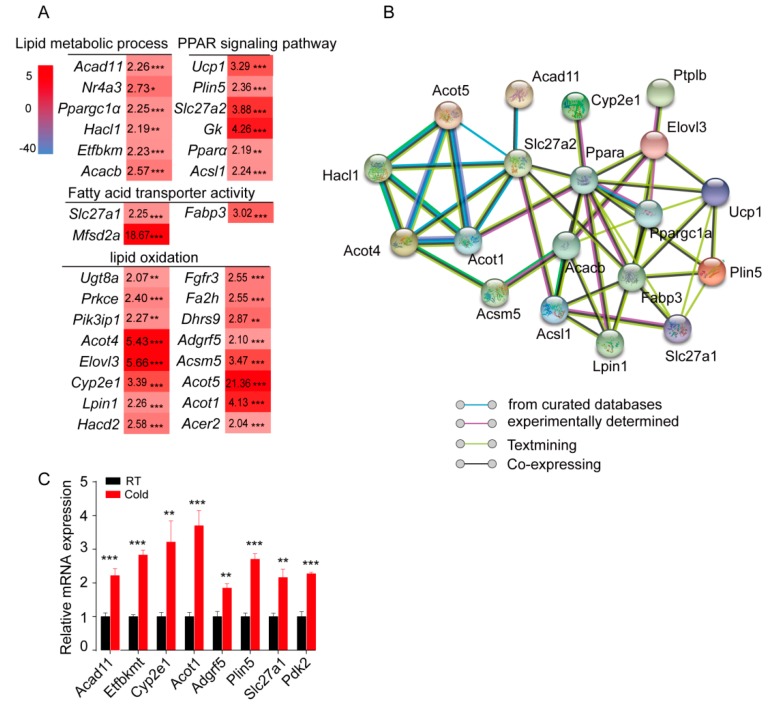
Acute cold exposure significantly alters the expression of genes involved in fatty acid metabolic processes and lipid oxidation. (**A**) Based on the RNA-sequencing data, the heat map was constructed from a panel of 31 upregulated genes, which were annotated in the GO terms lipid metabolic process, fatty acid metabolic process, lipid oxidation, fatty acid oxidation, and PPAR signaling pathway. * *p* < 0.05, ** *p* < 0.01 and *** *p* < 0.001 for differences between control and cold treatment groups. (**B**) Thirty-one genes were used to build the molecular network. (**C**) QPCR validation of randomly selected DEGs. ** *p* < 0.01 and *** *p* < 0.001 for differences between control and cold treatment groups.

A panel of 31 upregulated genes, which were involved in lipid metabolic processes (*Acad11*, *Nr4a3*, *Ppargc1a*, *Acacb*, etc.), lipid oxidation (*Ugt8a*, *Acot4*, *Elovl3*, *Acot1*, etc.), fatty acid transporter activity (*Slc27a1*, *Fabp3*, and *Mfsd2a*), and the PPAR signaling pathway (*Ucp1*, *Plin5*, *Slc27a2*, *Gk*, *Ppara*, and *Acsl1*), was profiled based on the RNA-Seq data (Figure 3A). Conversely, the expression of genes involved in the regulation of immune response (*Tnfrsf13c*, *Cd48*, *Ighd*, etc.), regulation of response to stimulus (*Npas2*, *Bank1*, *Gpr183*, etc.), cell adhesion (*Ncan*, *Sell*, *Egr3*, etc.), and defense response (*Hck*, *Blk*, *Ccr6*, etc.) was significantly downregulated in sWAT from cold-treated mice (Supplementary Figure S1A). In addition, the signaling network of these genes was analyzed with the online tool (https://string-db.org/), and the results showed that these genes were closely linked (Figure 3B). Furthermore, eight upregulated and six downregulated genes were randomly selected for further confirmation using QPCR, and all of them showed the same expression pattern as observed in RNA-seq (Figure 3C and Supplementary Figure S1B). 

### 2.4. Validation of the Potential WAT Browning Candidate Genes in 3T3-L1 Differentiation Toward Beige Adipocyte

**Figure 4:**
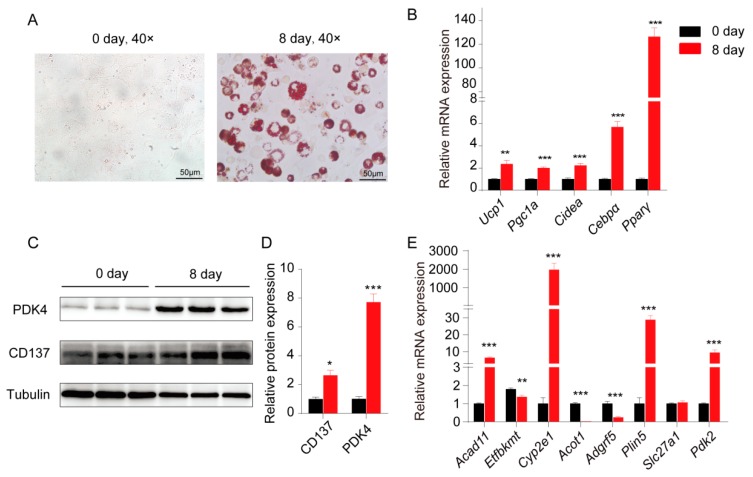
Gene expression was induced in 3T3-L1 cells during differentiation into beige adipocytes. (**A**) Oil Red O staining of 3T3-L1 cells undergoing beige adipogenic differentiation on days 0 and 8. (**B**) The relative mRNA expression of brown/beige adipocyte marker genes (*Ucp1*, *Pgc1a*, and *Cidea*) and adipogenic marker genes (*Cebpα* and *Pparγ*). (**C**) Immunoblot analysis of brown/ beige adipocyte marker proteins (PDK4 and CD137) in 3T3-L1 cells before and after differentiation, one representative blot from 3 independent experiments is shown. (**D**) Quantitative protein levels of PDK4 and CD137 from immunoblot. (**E**) The relative mRNA expression of genes in 3T3-L1 cells undergoing beige adipogenic differentiation on days 0 and 8. Results represent the mean ± SEM, * *p* < 0.05, ** *p* < 0.01 and ** *p* < 0.001 for differences between 3T3-L1 differentiation on 0 day and 8 day.

3T3-L1 cells were differentiated into beige adipocytes to further validate the induction of genes which might be involved in beige formation. Images of Oil Red O staining showed the high differentiation efficiency (Figure 4A), and lipid accumulation was further confirmed by the upregulation of BAT/beige marker genes (*Ucp1*, *Pgc1a*, and *Cidea*) and adipogenic marker genes (*Cebpα* and *Pparγ*) (Figure 4B). The levels of a BAT/beige marker, PDK4 and CD137, were detected in cells prior to and after differentiation using immunoblot, and the levels of both proteins were increased in the differentiated 3T3-L1 cells (Figure 4C). Quantitative data of immunoblot are shown in Figure 4D. All these data indicated that 3T3-L1 cells successfully differentiated into beige adipocytes. Next, we detected the expression levels of some genes that we identified in cold-stimulated sWAT, including *Acad11*, *Etfbkmt*, *Cyp2e1*, *Acot1*, *Adgrf5*, *Plin5*, *Slc27a1*, and *Pdk2*. Our data showed that four of these genes (*Acad11*, *Cyp2e1*, *Plin5*, and *Pdk2*) were significantly upregulated in differentiated 3T3-L1 cells, while *Etfbkmt*, *Acot1*, *and Adgrf5* were significantly downregulated (Figure 4E).

## 3. Discussion

It has been well-recognized that chronic cold treatment induces the formation of beige adipose tissue in mice [13]. Here, we show that acute cold exposure (4 °C for 4 h) could be able to induce WAT browning in mice, as evidenced by the presence of multilocular lipid droplets in H&E stained tissues and induction of the beige adipocyte marker CD137. This observation was consistent with our previous data, where WAT browning in mice was used as a control to investigate acute cold-stimulated WAT browning in pigs [14]. We are particularly interested in the changes in expression of genes involved in the acute cold-stimulated WAT browning process. Our data revealed 221 significantly upregulated genes that were enriched in the GO annotations of lipid metabolic process, fatty acid metabolic process, lipid oxidation, fatty acid oxidation, and the PPAR signaling pathway. These enriched GO terms were quite similar to the terms identified for DEGs in the inguinal WAT of chronic cold-treated (6 °C for 10 days) 129Sv mice [15]. Surprisingly, the GO terms of the downregulated genes were annotated as regulation of immune response, regulation of response to stimulus, cell adhesion, defense response, etc., which were distinct from those from chronic cold-treated 129Sv mice, which were 14-3-3 signaling, germ cell junction signal, lipid antigen presentation by CD1 and ER stress pathway, etc. [15]. This observation was also not consistent with our pig study, in which immune-related genes were upregulated in cold-treated sWAT of the Bama pig, a Chinese miniature pig breed [13]. The reasons for this discrepancy might be due to the different species, or strains, or different cold treatment conditions.

In our study, the GO term of lipid oxidation was significantly enriched in sWAT of mice after cold stimulation, which was consistent with the observation from chronic cold-treated sWAT from 129Sv mice [15] and acute cold-stimulated sWAT from Tibetan pigs, a well-recognized cold tolerant pig breed in China [14]. Fatty acid metabolic signals can be used as a marker of the white adipocyte browning process [16]. Specifically, we found that three genes from the acyl-CoA thioesterase (*Acots*) family, *Acot1*, *Acot4*, and *Acot5*, were significantly upregulated in cold-treated sWAT. This gene family contains 15 members and can be grouped into two subfamilies, structurally: type I *Acots* (*Acot*1–6, which contain the α/β-hydrolase domain) and type II *Acots* (*Acot*7–15, which contain the ‘Hotdog fold’ domain) [16]. ACOTs catalyze the conversion of acyl-CoAs to fatty acids and CoA and play a critical role in the regulation of fatty acid metabolism [17]. The function of type II *Acots* in thermogenesis has been studied extensively [16]; however, investigations of type I *Acots* in BAT/beige-mediated thermogenesis are quite sporadic. Similar to our results, *Acot1* was upregulated in cold-treated BAT in mice [18]. It has been reported that hepatic *Acot1* regulates *PPARα* expression, coupling fatty acid flux with oxidative capacity during fasting [19]. We also observed an upregulation of *PPARα* in cold-stimulated sWAT, which hinted at the possible regulatory role of *Acot1* on PPARα expression in cold-stimulated adipocytes. Elevation of *Acot4* expression was observed in the inguinal WAT of chronic cold-treated 129Sv mice [15], and there is no evidence showing that *Acot5* is involved in BAT/beige-mediated thermogenesis thus far. Taken together, our results showed that *Acot1*, *Acot4*, and *Acot5* were upregulated in cold-treated sWAT, but how they are involved in cold-induced WAT browning still needs to be further investigated.

PLIN5, a lipid droplet-associated protein, has been demonstrated to be an adipose triglyceride lipase partner [20] and provides physical and metabolic linkage to mitochondria [21,22,23]. Induction of *Plin5* in both sWAT of acute cold-treated mice and differentiated beige adipocytes revealed the involvement of ATGL-mediated lipolysis and mitochondrial-related cellular processes in cold-induced beige adipocyte formation. In addition, Rosell et al. screened a set of genes that define the “browning” of cold-activated WAT by studying the transcriptional response of adipose tissues to chronic cold exposure in 129Sv mice [15]. The overlapping genes identified by comparing this gene list with our upregulated genes, including *Fabp3*, *Pparα*, *Elovl3*, *Plin5*, and *Acot4*, might be valuable targets to further investigate the molecular basis of beige formation.

The activation and recruitment of thermogenic cells, such as BAT and beige adipocytes, has become a potential therapeutic target to counteract obesity and associated metabolic disorders. Despite that single cell resolution-based studies will be needed to identify the key regulators controlling beige formation due to the high heterogeneity of adipose tissues, we herein identified a set of candidate genes potentially required for cold-induced beige adipocyte biogenesis in mice. Further investigation of the roles of these genes in beige adipocyte formation will explore the possibility of therapeutic targets for obesity and its related metabolic diseases.

## 4. Materials and Methods

### 4.1. Animals and Sample Collection

Ten-week-old C57BL/6J male mice were purchased from Beijing Huafukang Bio-Technology Co., Ltd. Mice were group housed in individually ventilated cages within a barrier facility maintained at room temperature with 12 h light/dark cycles (7:00 a.m.–7:00 p.m.). When we began the cold challenge experiment, five mice were separated in individual cages at room temperature (22 ± 3 °C, RT) as a control group, and another five mice were housed individually as well at 4 °C for 4 h as the cold stimulation group (from 10:00 a.m. to 2:00 p.m.). All mice were fed a normal chow diet and had free access to water throughout the experiment. Animals were euthanized by cervical dislocation. Subcutaneous adipose tissue was dissected, frozen immediately in liquid nitrogen, and stored at −80 °C. All animal breeding procedures and experiments were approved by the Animal Ethics Committee of the Institute of Animal Science, Chinese Academy of Agricultural Sciences (10 September 2018, CAAS; approval no, IAS2018-2).

### 4.2. RNA Preparation and Quantitative Real-Time PCR

Tissues were lysed and homogenized in a TissueLyser (Qiagen, Hannover, Germany). Total RNA was extracted using TRIzol reagent (Invitrogen, Life Technologies, Grand Island, NY, USA) according to the manufacturer’s instructions. The quality and purity of total RNA were assessed using a microspectrophotometer (Nano-100, Nano Drop Technologies, Wilmington, DE, USA) and an Agilent 2100 Bioanalyzer (Agilent, CA, USA). The cDNA templates were synthesized from 2 µg of total RNA using a High Capacity cDNA Reverse Transcription Kit (Thermo Fisher Scientific, Waltham, MA, USA). The following reaction conditions were used: 42 °C for 60 min, followed by 70 °C for 5 min. QPCR was performed on three replicates of each sample using SYBR Green Master Mix (Applied Biosystems, Foster City, CA, USA) and a QuantStudio 3 instrument from Applied Biosystems (Thermo Fisher Scientific, Waltham, MA, USA). Reactions were incubated in a 96-well optical plate at 50 °C for 2 min, 95 °C for 2 min, and then 40 cycles of 95 °C for 15 s and 60 °C for 1 min. At the end of the PCR cycles, a melting curve analysis was performed to validate the specific generation of the expected PCR products. Relative expression levels of genes were calculated using the 2^-ΔΔCT^ method. The 18s rRNA gene was used as a housekeeping gene, and the primer sequences are listed in Supplementary Table S1.

### 4.3. RNA-Seq Analysis

Sequencing library preparation and RNA-seq analyses were conducted at Shanghai Personal Biotechnology Co., Ltd. RNA samples with high purity (OD 260/280 ≥ 2.0) and high integrity (RIN > 7) were used to construct the cDNA library. More detailed information on the cDNA library construction, sequencing of PE libraries, quality control, read mapping, and FPKM calculations is presented in a previous study [24]. Differentially expressed genes were defined using a threshold *p*-value < 0.05 and fold change > 2.

### 4.4. Gene Ontology (GO) and Signaling Network Analyses

The GO enrichment analysis of upregulated and downregulated genes was performed using the ‘DAVID 6.8′ Functional Annotation Tool (https://david.ncifcrf.gov/summary.jsp). GO terms with *p*-values < 0.05 were regarded as statistically significant. Signaling networks of differentially expressed genes were analyzed using an online tool (https://string-db.org/).

### 4.5. Hematoxylin and Eosin (H&E) Staining

Paraffin-embedded sWAT sections (5 µm) were stained with H&E as previously described [25]. The adipose tissues were fixed with 4% paraformaldehyde for 48 h. Fixed tissues were dehydrated in an ethanol gradient from 75% to 100% and embedded in paraffin. The embedded tissues were sectioned (5 µm), stained with hematoxylin and eosin (H&E, Beyotime Institute of Biology, Suzhou, China), and imaged using an EVOS XL Core cell imaging system (Life Technologies).

### 4.6. Western Blot Analysis

The adipose tissues were lysed in M-PER Mammalian Protein Extraction Reagent (Thermo Fisher Scientific, Waltham, MA, USA) supplemented with a protease inhibitor cocktail (Roche, Indianapolis, IN, USA). Total proteins (20–50 μg) were separated using 10% SDS-PAGE and transferred to PVDF membranes (Millipore, Madison, WI, USA). After being blocked in TBST with 5% skim milk for 2 h at room temperature, membranes were incubated with the primary antibody at 4 °C overnight. β-Tubulin (1:2000, CST, Danvers, MA, USA) was used as the loading control. Horseradish peroxidase-conjugated goat anti-rabbit IgG (1:5000) was used as a secondary antibody, and immunoreactive bands were detected using a FluorChem M Fluorescent Imaging System (Tanon 5200, Tanon Science & Technology Co., Ltd., Shanghai, China) with Pierce Enhanced Chemiluminescence (ECL) Western Blotting Substrate (Thermo Scientific Pierce, Rockford, IL, USA). The primary antibodies used in the present study included CD137 (1:1000, Bioss, Beijing, China) and PDK4 (1:1000, Abcam, Cambridge, UK).

### 4.7. Cell Culture and in Vitro Browning

Mouse 3T3-L1 preadipocytes were obtained from Peking Union Medical College Hospital (Beijing, China) and incubated at 37 °C in a humidified 5% CO_2_ atmosphere in DMEM (Lonza, Switzerland) containing 10% fetal bovine serum (FBS) and 1% penicillin-streptomycin. Upon confluence (day 0), the media was replaced with adipogenic differentiation medium (DM) containing 10% fetal bovine serum (FBS), 20 nM insulin, 0.5 mM 3-isobutyl-1-methylxanthine (IBMX), 2 µM dexamethasone (DEX), 1 nM T3, 0.125 mM indomethacin, and 1 µM rosiglitazone. The culture was incubated at 37 °C in 5% CO_2_ for 4 days. The media was then replaced with DMEM supplemented with 10% FBS, 20 nM insulin, and 1 nM T3 for another 4 days. After differentiation, cells were harvested for the further use.

### 4.8. Oil Red O Staining

Intracellular lipid accumulation was visualized by Oil Red O (Solarbio, Beijing, China) staining on day 8 [26]. Briefly, the cells were washed with DPBS and fixed with 4% paraformaldehyde for 15 min at room temperature. Then, the cells were washed with distilled water, 60% isopropanol, and stained with Oil Red O solution (60% isopropanol in water) for 15 min at room temperature. After rinsing with distilled water and 60% isopropanol, the cells were photographed under a microscope (Leica, Wetzlar, Germany).

### 4.9. Statistical Analysis

Statistical analyses between two groups were performed using two-tailed and unpaired Student’s *t*-test. All data are reported as the means ±SEM. The statistical significance of differences between groups was denoted as * *p* < 0.05, ** *p* < 0.01, and *** *p*< 0.001. 

## Supplementary Materials

Supplementary materials can be found at https://www.mdpi.com/1422-0067/20/16/3968/s1.

## Abbreviations

sWAT	subcutaneous white adipose tissues
BAT	brown adipose tissue
WAT	white adipose tissue
DEGs	differentially expressed genes
GO	gene ontology
RT	room temperature
QPCR	quantitative real-time PCR
PPARγ	peroxisome proliferator-activated receptor-γ
