# Supplementary material for: Transcriptional Response of Subcutaneous White Adipose Tissue to Acute Cold Exposure in Mice
Source: Int J Mol Sci. 2019 Aug 15;20(16):3968. doi: 10.3390/ijms20163968 (PMC6720191; doi:10.3390/ijms20163968)
Supplement: Supplementary file 1 [file ijms-20-03968-s001.zip › Supplementary files/Supplementary Figure S1.docx]

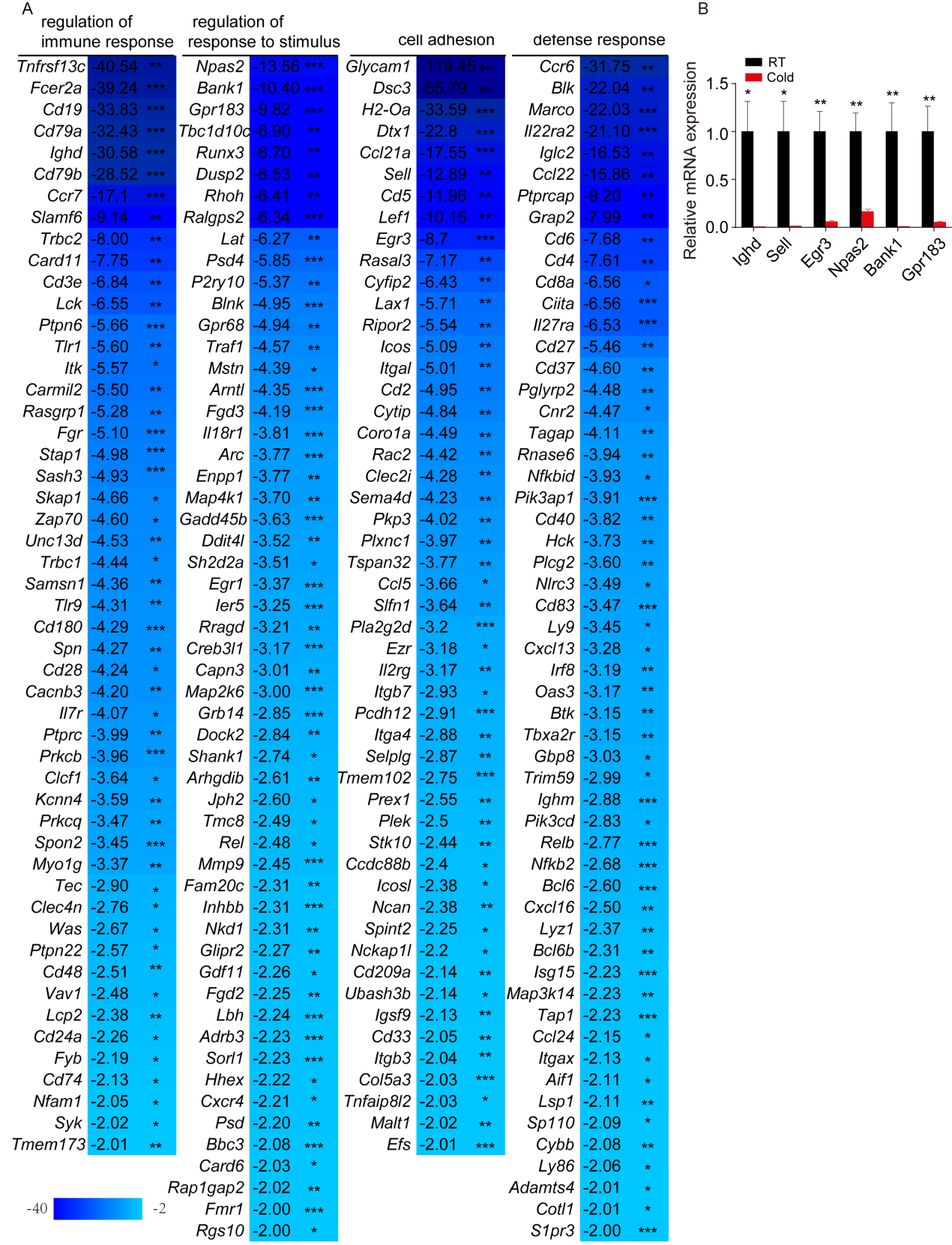


Supplementary figure S1. Acute cold exposure significantly alters the expression of genes involved in regulation of immune response and regulation of response to stimulus. (A) Based on the RNA-sequencing data, the heat map was constructed from a panel of 212 downregulated genes, which were annotated in the GO terms regulation of immune response, regulation of response to stimulus, cell adhesion and defense response. (B) QPCR validation of randomly selected genes shown in (A). **p*<0.05, ***p*<0.01 and ****p*<0.001 for differences between RT and cold stimulation.
