# Supplementary material for: Transcriptional Response of Subcutaneous White Adipose Tissue to Acute Cold Exposure in Mice
Source: Int J Mol Sci. 2019 Aug 15;20(16):3968. doi: 10.3390/ijms20163968 (PMC6720191; doi:10.3390/ijms20163968)
Supplement: Supplementary file 1 [file ijms-20-03968-s001.zip › Supplementary files/Supplementary Tables.docx]

Supporting information

Table S1 Primers for QPCR

| Gene | Primer Name | Primer Sequence (5′-3′) |
| --- | --- | --- |
| *Ucp1* | Forward | ACTGCCACACCTCCAGTCATT |
|  | Reversed | CTTTGCCTCACTCAGGATTGG |
| *Prdm16* | Forward | CAGCACGGTGAAGCCATTC |
|  | Reversed | GGCGTGCATCCGCTTGTGC |
| *PGC1α* | Forward | CCCTGCCATTGTTAAGACC |
|  | Reversed | TGCTGCTGTTCCTGTTTTC |
| *Cidea* | Forward | ATCACAACTGGCCTGGTTACG |
|  | Reversed | TACTACCCGGTGTCCATTTCT |
| *Elovl3* | Forward | TGTTGGCCAGACCTACATGA |
|  | Reversed | ATCCGTGTAGATGGCAAAGC |
| *Acad11* | Forward | GTGCTACCCCAGCACAAGTT |
|  | Reversed | TGCTTTGGGAAGAAGAGAGC |
| *Etfbkmt* | Forward | AACGACATAGACCCCATTGC |
|  | Reversed | GTTCTGCAGCCACAGATGAA |
| *Cyp2e1* | Forward | AGGCTGTCAAGGAGGTGCTA |
|  | Reversed | GGAAGTGTGCCTCTCTTTGG |
| *Acot1* | Forward | TTTTTGGCGATTGGTCAAG |
|  | Reversed | CAGGAAAGGGTCCAGGTTCT |
| *Adgrf5* | Forward | GTGGCGGAAGAATACATGGT |
|  | Reversed | TCCTCAGACCACTGGTAGCC |
| *Plin5* | Forward | CCTTGCTGAGCACTGTGTGT |
|  | Reversed | GCTGATGTCACCACCATGTC |
| *Slc27a1* | Forward | TTCTGCGTATCGTCTGCAAG |
|  | Reversed | GCGAAGGTCCAGCATATACC |
| *Pdk2* | Forward | AGACTTCGGATCCAGCAATG |
|  | Reversed | TTCCATGATGTCCAGCAGAC |
| *Ighd* | Forward | CAATGGTCCTCCAGGTCACT |
|  | Reversed | ATGGCCCCTTCTATGTCCTT |
| *Sell* | Forward | CTGAGGCCTGCAGAGAGACT |
|  | Reversed | AGCATTTTCCCAGTTCATGG |
| *Egr3* | Forward | CAATCTGTACCCCGAGGAGA |
|  | Reversed | TCCCAAGTAGGTCACGGTCT |
| *Npas2* | Forward | CCATGCTCCCTGGTAACACT |
|  | Reversed | GAAGCCATCTAATGCCTCCA |
| *Bank1* | Forward | AAAGGTCTGCAGGGGAAGTT |
|  | Reversed | TGGCTCTTGAGTCTGTTGGA |
| *Gpr183* | Forward | TTCTGAGCAAACACGGACTG |
|  | Reversed | ACCAGCCCAATGATGAAGAC |
| *C/EBPα* | Forward | GTTAGCCATGTGGTAGGAGACA |
|  | Reversed | CCCAGCCGTTAGTGAAGAGT |
| *PPARγ* | Forward | ACCACTCGCATTCCTTTGAC |
|  | Reversed | TGGGTCAGCTCTTGTGAATG |
| *18s rRNA* | Forward | GTAACCCGTTGAACCCCATT |
|  | Reversed | CCATCCAATCGGTAGTAGCG |

Table S2 Upregulated genes (*p*<0.05 and log2FC >1)

| No. | Gene_ID | Gene Name | WT(FPKM) | KI(FPKM) | log2FC | *p*_value |
| --- | --- | --- | --- | --- | --- | --- |
| 1 | ENSMUSG00000028341 | *NR4A3* | 378.5 | 1052 | 1.45 | 4.07E-02 |
| 2 | ENSMUSG00000003477 | *Inmt* | 3261.25 | 8766 | 1.02 | 2.67E-02 |
| 3 | ENSMUSG00000052316 | *LRRC15* | 46 | 96 | 1.02 | 1.29E-02 |
| 4 | ENSMUSG00000008307 | *1700109H08RIK* | 38.25 | 91.5 | 1.02 | 9.64E-03 |
| 5 | ENSMUSG00000089756 | *ZFP966* | 106.5 | 225.5 | 1.02 | 8.40E-03 |
| 6 | ENSMUSG00000028654 | *MYCL* | 184.25 | 480.5 | 1.04 | 8.18E-03 |
| 7 | ENSMUSG00000022510 | *TRP63* | 154 | 358.5 | 1.02 | 8.08E-03 |
| 8 | ENSMUSG00000003526 | *Prodh* | 55 | 135.75 | 1.01 | 7.89E-03 |
| 9 | ENSMUSG00000021367 | *EDN1* | 147 | 414.75 | 1.16 | 6.87E-03 |
| 10 | ENSMUSG00000020593 | *Lpin1* | 40.5 | 94.25 | 1.00 | 6.06E-03 |
| 11 | ENSMUSG00000046215 | *RPRML* | 78.5 | 227 | 1.23 | 5.30E-03 |
| 12 | ENSMUSG00000097324 | *CARMN* | 60.75 | 143.25 | 1.01 | 4.99E-03 |
| 13 | ENSMUSG00000068950 | *OLFR338* | 40.5 | 110.5 | 1.25 | 4.46E-03 |
| 14 | ENSMUSG00000027068 | *DHRS9* | 72.25 | 265.75 | 1.52 | 4.44E-03 |
| 15 | ENSMUSG00000021260 | *Hhipl1* | 52.25 | 139.75 | 1.23 | 4.03E-03 |
| 16 | ENSMUSG00000027313 | *CHAC1* | 65.75 | 172 | 1.11 | 3.71E-03 |
| 17 | ENSMUSG00000037440 | *VNN1* | 185.25 | 510.75 | 1.11 | 3.59E-03 |
| 18 | ENSMUSG00000036745 | *TTLL7* | 116.25 | 275.75 | 1.02 | 3.21E-03 |
| 19 | ENSMUSG00000046167 | *GLDN* | 33.75 | 86.75 | 1.20 | 3.02E-03 |
| 20 | ENSMUSG00000097113 | *GM19705* | 35.75 | 82.75 | 1.07 | 2.39E-03 |
| 21 | ENSMUSG00000021884 | *Hacl1* | 37 | 87 | 1.08 | 2.16E-03 |
| 22 | ENSMUSG00000097462 | *9530026P05RIK* | 46.75 | 135.25 | 1.30 | 2.10E-03 |
| 23 | ENSMUSG00000022383 | *Ppara* | 72.5 | 178.75 | 1.13 | 2.09E-03 |
| 24 | ENSMUSG00000097494 | *4933406C10RIK* | 49 | 129 | 1.19 | 2.04E-03 |
| 25 | ENSMUSG00000074971 | *FIBIN* | 91 | 217.25 | 1.16 | 1.95E-03 |
| 26 | ENSMUSG00000042359 | *OSBPL6* | 116.25 | 266.75 | 1.01 | 1.92E-03 |
| 27 | ENSMUSG00000034614 | *PIK3IP1* | 249 | 692.5 | 1.19 | 1.84E-03 |
| 28 | ENSMUSG00000023153 | *Tmem52* | 53.75 | 148.75 | 1.21 | 1.79E-03 |
| 29 | ENSMUSG00000025059 | *Gk* | 396.25 | 1109 | 1.21 | 1.77E-03 |
| 30 | ENSMUSG00000025437 | *Usp33* | 469.75 | 1177.25 | 1.13 | 1.75E-03 |
| 31 | ENSMUSG00000032854 | *UGT8A* | 89.5 | 216 | 1.05 | 1.73E-03 |
| 32 | ENSMUSG00000025479 | *Cyp2e1* | 37 | 92.5 | 1.18 | 1.39E-03 |
| 33 | ENSMUSG00000112980 | *CT030173.1* | 48.25 | 153.25 | 1.40 | 1.14E-03 |
| 34 | ENSMUSG00000026442 | *NFASC* | 84 | 204.5 | 1.08 | 1.10E-03 |
| 35 | ENSMUSG00000042453 | *RELN* | 94.75 | 220 | 1.01 | 1.06E-03 |
| 36 | ENSMUSG00000025515 | *Muc2* | 42 | 180.5 | 1.64 | 1.01E-03 |
| 37 | ENSMUSG00000025930 | *MSC* | 45.5 | 139.5 | 1.40 | 9.77E-04 |
| 38 | ENSMUSG00000103965 | *GM30173* | 48 | 124.25 | 1.21 | 8.02E-04 |
| 39 | ENSMUSG00000033871 | *PPARGC1B* | 381 | 897.75 | 1.01 | 7.39E-04 |
| 40 | ENSMUSG00000027359 | *Slc27a2* | 180.75 | 641.5 | 1.55 | 6.62E-04 |
| 41 | ENSMUSG00000062329 | *CYTL1* | 89.5 | 225.75 | 1.19 | 5.87E-04 |
| 42 | ENSMUSG00000007480 | *MC5R* | 34.75 | 138.75 | 1.60 | 5.59E-04 |
| 43 | ENSMUSG00000100813 | *GM28874* | 40.5 | 106 | 1.27 | 5.50E-04 |
| 44 | ENSMUSG00000028773 | *Fabp3* | 210.25 | 491.25 | 1.01 | 4.98E-04 |
| 45 | ENSMUSG00000029167 | *Ppargc1a* | 150.5 | 384.5 | 1.14 | 4.43E-04 |
| 46 | ENSMUSG00000041361 | *MYZAP* | 61.25 | 161.75 | 1.24 | 4.41E-04 |
| 47 | ENSMUSG00000021699 | *PDE4D* | 670.5 | 1572.25 | 1.13 | 4.18E-04 |
| 48 | ENSMUSG00000026796 | *FAM129B* | 858.75 | 2409.5 | 1.34 | 4.11E-04 |
| 49 | ENSMUSG00000031842 | *PDE4C* | 94 | 224 | 1.12 | 3.78E-04 |
| 50 | ENSMUSG00000038917 | *3930402G23RIK* | 36.25 | 115.5 | 1.55 | 3.75E-04 |
| 51 | ENSMUSG00000048572 | *TMEM252* | 98.25 | 238.5 | 1.10 | 3.69E-04 |
| 52 | ENSMUSG00000019990 | *PDE7B* | 415.75 | 1062 | 1.26 | 3.54E-04 |
| 53 | ENSMUSG00000029228 | *Lnx1* | 971.75 | 2261.75 | 1.17 | 3.35E-04 |
| 54 | ENSMUSG00000029321 | *Slc10a6* | 23 | 122 | 2.05 | 3.06E-04 |
| 55 | ENSMUSG00000053198 | *PRX* | 646 | 1501.75 | 1.01 | 3.04E-04 |
| 56 | ENSMUSG00000029778 | *Adcyap1r1* | 90 | 224.5 | 1.10 | 3.01E-04 |
| 57 | ENSMUSG00000030671 | *Pde3b* | 51.75 | 141.5 | 1.38 | 2.87E-04 |
| 58 | ENSMUSG00000054252 | *FGFR3* | 107.75 | 298.25 | 1.35 | 2.83E-04 |
| 59 | ENSMUSG00000059824 | *DBP* | 1099.75 | 3433.25 | 1.28 | 2.78E-04 |
| 60 | ENSMUSG00000091304 | *SPEER6-PS1* | 24 | 125.25 | 2.23 | 2.75E-04 |
| 61 | ENSMUSG00000032186 | *TMOD2* | 259.5 | 629.5 | 1.04 | 2.74E-04 |
| 62 | ENSMUSG00000078486 | *PERM1* | 126 | 386.75 | 1.27 | 2.57E-04 |
| 63 | ENSMUSG00000032656 | *MARCH3* | 303 | 692.75 | 1.00 | 2.50E-04 |
| 64 | ENSMUSG00000033610 | *PANK1* | 737.75 | 1862.75 | 1.18 | 2.44E-04 |
| 65 | ENSMUSG00000020431 | *ADCY1* | 64.75 | 173.75 | 1.27 | 2.06E-04 |
| 66 | ENSMUSG00000030889 | *VWA3A* | 416 | 994 | 1.04 | 2.01E-04 |
| 67 | ENSMUSG00000000223 | *DRP2* | 426 | 1021.25 | 1.13 | 1.90E-04 |
| 68 | ENSMUSG00000107655 | *GM44220* | 80 | 203.25 | 1.21 | 1.79E-04 |
| 69 | ENSMUSG00000108368 | *GM45053* | 32.75 | 97.25 | 1.32 | 1.78E-04 |
| 70 | ENSMUSG00000045180 | *SHROOM2* | 54 | 140.75 | 1.25 | 1.76E-04 |
| 71 | ENSMUSG00000020435 | *OSBP2* | 75 | 225 | 1.32 | 1.75E-04 |
| 72 | ENSMUSG00000022468 | *ENDOU* | 155.75 | 431.25 | 1.31 | 1.66E-04 |
| 73 | ENSMUSG00000020038 | *CRY1* | 251.5 | 596.25 | 1.17 | 1.56E-04 |
| 74 | ENSMUSG00000027375 | *MAL* | 301.5 | 722.25 | 1.09 | 1.53E-04 |
| 75 | ENSMUSG00000031596 | *Slc7a2* | 111.5 | 351.75 | 1.37 | 1.17E-04 |
| 76 | ENSMUSG00000031298 | *ADGRG2* | 385.25 | 1021.75 | 1.15 | 1.15E-04 |
| 77 | ENSMUSG00000054582 | *PABPC1L* | 91.25 | 357.75 | 1.91 | 1.05E-04 |
| 78 | ENSMUSG00000037411 | *SERPINE1* | 171 | 647.75 | 1.85 | 9.98E-05 |
| 79 | ENSMUSG00000031765 | *Mt1* | 54.75 | 146.5 | 1.31 | 9.47E-05 |
| 80 | ENSMUSG00000043398 | *GPR135* | 250.75 | 624.25 | 1.10 | 9.30E-05 |
| 81 | ENSMUSG00000045392 | *OLFR1033* | 89.5 | 352.75 | 1.89 | 9.02E-05 |
| 82 | ENSMUSG00000032578 | *Cish* | 77 | 224.5 | 1.27 | 8.87E-05 |
| 83 | ENSMUSG00000038754 | *Elovl3* | 199.5 | 493 | 1.23 | 8.19E-05 |
| 84 | ENSMUSG00000020905 | *USP43* | 38.5 | 131.5 | 1.62 | 8.17E-05 |
| 85 | ENSMUSG00000039958 | *ETFBKMT* | 609 | 1545.75 | 1.16 | 7.30E-05 |
| 86 | ENSMUSG00000040170 | *Fmo2* | 27 | 159.5 | 2.26 | 6.90E-05 |
| 87 | ENSMUSG00000021702 | *THBS4* | 24.5 | 119.75 | 1.90 | 6.56E-05 |
| 88 | ENSMUSG00000031710 | *UCP1* | 8833 | 32559 | 1.72 | 4.97E-05 |
| 89 | ENSMUSG00000057913 | *GM10032* | 36.25 | 205.75 | 2.34 | 4.15E-05 |
| 90 | ENSMUSG00000038007 | *ACER2* | 1769.25 | 3821.5 | 1.03 | 4.00E-05 |
| 91 | ENSMUSG00000101389 | *MS4A4A* | 148.25 | 470.25 | 1.37 | 3.56E-05 |
| 92 | ENSMUSG00000033579 | *FA2H* | 82.25 | 249.5 | 1.35 | 3.17E-05 |
| 93 | ENSMUSG00000014602 | *KIF1A* | 79.5 | 267.5 | 1.46 | 2.99E-05 |
| 94 | ENSMUSG00000035873 | *PAWR* | 78.5 | 224 | 1.36 | 2.94E-05 |
| 95 | ENSMUSG00000041592 | *Sdk2* | 294.75 | 1095.25 | 1.59 | 2.69E-05 |
| 96 | ENSMUSG00000043155 | *Hpdl* | 5216.25 | 11583.25 | 1.02 | 2.51E-05 |
| 97 | ENSMUSG00000060961 | *SLC4A4* | 758.25 | 2579.75 | 1.49 | 2.19E-05 |
| 98 | ENSMUSG00000048787 | *DCUN1D3* | 1572 | 3514.25 | 1.02 | 1.98E-05 |
| 99 | ENSMUSG00000091575 | *2010016I18RIK* | 444.25 | 1888.75 | 1.81 | 1.64E-05 |
| 100 | ENSMUSG00000028776 | *TINAGL1* | 2060 | 5211.25 | 1.20 | 1.56E-05 |
| 101 | ENSMUSG00000043498 | *9330132A10Rik* | 308 | 798.75 | 1.22 | 1.50E-05 |
| 102 | ENSMUSG00000100627 | *A830008E24RIK* | 43.5 | 134.75 | 1.43 | 1.44E-05 |
| 103 | ENSMUSG00000067201 | *H2-M9* | 64.5 | 191.5 | 1.39 | 1.27E-05 |
| 104 | ENSMUSG00000018417 | *MYO1B* | 1048 | 2429 | 1.08 | 1.07E-05 |
| 105 | ENSMUSG00000017009 | *SDC4* | 781 | 2713.5 | 1.59 | 9.79E-06 |
| 106 | ENSMUSG00000044244 | *Il20rb* | 121 | 871.25 | 2.50 | 9.65E-06 |
| 107 | ENSMUSG00000027074 | *SLC43A3* | 2400 | 5903.25 | 1.05 | 9.40E-06 |
| 108 | ENSMUSG00000022622 | *ACR* | 35.5 | 124 | 1.66 | 8.27E-06 |
| 109 | ENSMUSG00000096954 | *GDAP10* | 22.25 | 103 | 2.01 | 6.55E-06 |
| 110 | ENSMUSG00000025981 | *COQ10B* | 805.5 | 2039.5 | 1.24 | 5.51E-06 |
| 111 | ENSMUSG00000047205 | *DUSP18* | 545.5 | 1454.5 | 1.16 | 5.49E-06 |
| 112 | ENSMUSG00000021067 | *SAV1* | 1291 | 3161.75 | 1.11 | 5.06E-06 |
| 113 | ENSMUSG00000011305 | *PLIN5* | 957.25 | 2526.5 | 1.24 | 5.05E-06 |
| 114 | ENSMUSG00000047216 | *Cdh19* | 40 | 141.25 | 1.71 | 4.13E-06 |
| 115 | ENSMUSG00000048489 | *Depp1* | 171.5 | 814.5 | 1.95 | 4.02E-06 |
| 116 | ENSMUSG00000050777 | *Tmem37* | 931.5 | 2394 | 1.21 | 3.75E-06 |
| 117 | ENSMUSG00000045973 | *SLC25A51* | 6646 | 14814 | 1.02 | 3.48E-06 |
| 118 | ENSMUSG00000068794 | *COL28A1* | 123 | 369 | 1.42 | 3.40E-06 |
| 119 | ENSMUSG00000043924 | *NCMAP* | 73.5 | 246.5 | 1.53 | 3.08E-06 |
| 120 | ENSMUSG00000072949 | *ACOT1* | 34 | 167 | 2.05 | 2.89E-06 |
| 121 | ENSMUSG00000052273 | *Dnah3* | 49.5 | 164 | 1.65 | 2.76E-06 |
| 122 | ENSMUSG00000026082 | *REV1* | 283.25 | 751.5 | 1.19 | 2.52E-06 |
| 123 | ENSMUSG00000052392 | *Acot4* | 33.25 | 136 | 1.85 | 2.17E-06 |
| 124 | ENSMUSG00000003153 | *SLC2A3* | 238 | 1089.25 | 1.88 | 1.76E-06 |
| 125 | ENSMUSG00000062563 | *CYS1* | 73.5 | 310.25 | 1.86 | 1.73E-06 |
| 126 | ENSMUSG00000019970 | *SGK1* | 1705 | 4151.75 | 1.11 | 1.23E-06 |
| 127 | ENSMUSG00000063406 | *Tmed5* | 760.75 | 1943.75 | 1.13 | 1.14E-06 |
| 128 | ENSMUSG00000064105 | *Cnnm2* | 1332.75 | 3167.75 | 1.05 | 1.01E-06 |
| 129 | ENSMUSG00000024899 | *PAPSS2* | 2079.5 | 4872.75 | 1.06 | 8.92E-07 |
| 130 | ENSMUSG00000020044 | *TIMP3* | 3969 | 9304.75 | 1.05 | 8.56E-07 |
| 131 | ENSMUSG00000026692 | *FMO4* | 64.5 | 228.5 | 1.60 | 6.87E-07 |
| 132 | ENSMUSG00000007682 | *DIO2* | 1107.25 | 6965 | 2.44 | 6.61E-07 |
| 133 | ENSMUSG00000064369 | *mt-Te* | 180 | 580.5 | 1.55 | 6.24E-07 |
| 134 | ENSMUSG00000069919 | *Hba-a1* | 169.25 | 504.5 | 1.47 | 5.81E-07 |
| 135 | ENSMUSG00000074899 | *Sptbn5* | 11354.25 | 26009 | 1.04 | 4.88E-07 |
| 136 | ENSMUSG00000072964 | *BHLHB9* | 722.25 | 2420 | 1.51 | 4.75E-07 |
| 137 | ENSMUSG00000040111 | *GRAMD1B* | 2125.75 | 5784.25 | 1.19 | 4.74E-07 |
| 138 | ENSMUSG00000027966 | *COL11A1* | 38.75 | 172.5 | 1.96 | 4.48E-07 |
| 139 | ENSMUSG00000042401 | *CRTAC1* | 49.25 | 280.25 | 2.31 | 4.48E-07 |
| 140 | ENSMUSG00000078866 | *Zfp970* | 37855.5 | 169714.75 | 1.76 | 3.84E-07 |
| 141 | ENSMUSG00000019997 | *CTGF* | 123.25 | 442.75 | 1.72 | 3.63E-07 |
| 142 | ENSMUSG00000020893 | *PER1* | 2388.75 | 6271 | 1.18 | 3.13E-07 |
| 143 | ENSMUSG00000056492 | *ADGRF5* | 5871.5 | 13156 | 1.07 | 2.94E-07 |
| 144 | ENSMUSG00000048355 | *ARXES1* | 1528.25 | 3656.75 | 1.07 | 2.36E-07 |
| 145 | ENSMUSG00000030483 | *CYP2B10* | 18 | 117.75 | 2.50 | 2.25E-07 |
| 146 | ENSMUSG00000031442 | *MCF2L* | 963.75 | 2822.5 | 1.34 | 1.99E-07 |
| 147 | ENSMUSG00000006641 | *SLC5A6* | 1267.5 | 3909.25 | 1.33 | 1.53E-07 |
| 148 | ENSMUSG00000083111 | *Gm14421* | 540 | 3022.25 | 2.32 | 1.33E-07 |
| 149 | ENSMUSG00000070495 | *CTCFL* | 275.5 | 801 | 1.35 | 1.25E-07 |
| 150 | ENSMUSG00000031808 | *SLC27A1* | 24281.75 | 66539.25 | 1.17 | 1.21E-07 |
| 151 | ENSMUSG00000042010 | *ACACB* | 13266.5 | 40049.25 | 1.36 | 1.14E-07 |
| 152 | ENSMUSG00000084512 | *Gm22482* | 126.25 | 448.75 | 1.60 | 1.10E-07 |
| 153 | ENSMUSG00000086320 | *Gm12840* | 92.75 | 341.25 | 1.65 | 9.85E-08 |
| 154 | ENSMUSG00000054555 | *ADAM12* | 1964.5 | 5894.5 | 1.30 | 7.95E-08 |
| 155 | ENSMUSG00000087475 | *4933406I18Rik* | 16 | 130 | 2.88 | 7.69E-08 |
| 156 | ENSMUSG00000091119 | *Ccdc152* | 46 | 185.25 | 1.85 | 7.60E-08 |
| 157 | ENSMUSG00000094786 | *Gm14403* | 277 | 2090.5 | 2.87 | 7.48E-08 |
| 158 | ENSMUSG00000018846 | *PANK3* | 11765 | 27758.75 | 1.01 | 7.27E-08 |
| 159 | ENSMUSG00000090150 | *ACAD11* | 3005.5 | 7898.5 | 1.18 | 7.07E-08 |
| 160 | ENSMUSG00000009394 | *SYN2* | 239.75 | 731 | 1.45 | 6.06E-08 |
| 161 | ENSMUSG00000097379 | *Gm26873* | 50.25 | 205 | 1.87 | 5.83E-08 |
| 162 | ENSMUSG00000097970 | *Gm27028* | 78.25 | 285.25 | 1.74 | 5.26E-08 |
| 163 | ENSMUSG00000045038 | *PRKCE* | 1550 | 4009.75 | 1.27 | 4.39E-08 |
| 164 | ENSMUSG00000032898 | *FBXO21* | 4014.5 | 10329.25 | 1.20 | 3.05E-08 |
| 165 | ENSMUSG00000033863 | *KLF9* | 2413.25 | 5944 | 1.13 | 2.95E-08 |
| 166 | ENSMUSG00000032311 | *NRG4* | 2393 | 6125.5 | 1.15 | 2.13E-08 |
| 167 | ENSMUSG00000004328 | *HIF3A* | 53.25 | 295.5 | 2.21 | 1.69E-08 |
| 168 | ENSMUSG00000031016 | *WEE1* | 128 | 451 | 1.63 | 1.21E-08 |
| 169 | ENSMUSG00000098708 | *Gm27252* | 10373 | 26726.5 | 1.18 | 1.20E-08 |
| 170 | ENSMUSG00000033453 | *ADAMTS15* | 648.75 | 2142 | 1.45 | 9.79E-09 |
| 171 | ENSMUSG00000097762 | *4732463B04RIK* | 27.5 | 180.75 | 2.59 | 8.39E-09 |
| 172 | ENSMUSG00000031431 | *TSC22D3* | 2494.5 | 7122.75 | 1.27 | 7.74E-09 |
| 173 | ENSMUSG00000041794 | *MYRIP* | 103.75 | 481.5 | 2.01 | 6.43E-09 |
| 174 | ENSMUSG00000101111 | *Gm28437* | 2005.25 | 9215 | 2.12 | 5.93E-09 |
| 175 | ENSMUSG00000030972 | *ACSM5* | 252.25 | 1012 | 1.80 | 3.99E-09 |
| 176 | ENSMUSG00000028982 | *SLC25A33* | 167.5 | 624 | 1.73 | 3.74E-09 |
| 177 | ENSMUSG00000048040 | *ARXES2* | 3882.5 | 10880.25 | 1.30 | 3.67E-09 |
| 178 | ENSMUSG00000109904 | *GM45819* | 24.25 | 151.75 | 2.51 | 3.64E-09 |
| 179 | ENSMUSG00000020848 | *DOC2B* | 37.5 | 285.75 | 2.62 | 3.50E-09 |
| 180 | ENSMUSG00000025902 | *SOX17* | 429 | 1336.25 | 1.52 | 3.42E-09 |
| 181 | ENSMUSG00000103047 | *Gm37310* | 720.5 | 2521.25 | 1.55 | 2.91E-09 |
| 182 | ENSMUSG00000032786 | *ALAS1* | 6495 | 16044 | 1.11 | 2.52E-09 |
| 183 | ENSMUSG00000020108 | *DDIT4* | 615 | 2488.5 | 1.71 | 2.16E-09 |
| 184 | ENSMUSG00000018796 | *ACSL1* | 81964.25 | 210583.75 | 1.17 | 1.97E-09 |
| 185 | ENSMUSG00000097815 | *GM26809* | 4129.25 | 17239.25 | 1.92 | 1.79E-09 |
| 186 | ENSMUSG00000104350 | *Gm38244* | 1222.5 | 5954 | 2.09 | 7.58E-10 |
| 187 | ENSMUSG00000074794 | *ARRDC3* | 1024 | 3615.5 | 1.54 | 3.52E-10 |
| 188 | ENSMUSG00000003949 | *HLF* | 422.5 | 1751.25 | 1.83 | 3.27E-10 |
| 189 | ENSMUSG00000020654 | *ADCY3* | 931.5 | 3506 | 1.72 | 3.15E-10 |
| 190 | ENSMUSG00000105245 | *Gm31305* | 629.25 | 2076.75 | 1.62 | 3.06E-10 |
| 191 | ENSMUSG00000030278 | *CIDEC* | 43878.75 | 125009.75 | 1.26 | 2.48E-10 |
| 192 | ENSMUSG00000108825 | *Gm45838* | 33 | 2017.25 | 5.99 | 2.00E-10 |
| 193 | ENSMUSG00000021903 | *GALNT15* | 1624 | 5416.75 | 1.51 | 2.64E-11 |
| 194 | ENSMUSG00000030495 | *SLC7A10* | 4301 | 13003.5 | 1.33 | 1.40E-11 |
| 195 | ENSMUSG00000110170 | *St6galnac2* | 122 | 725 | 2.33 | 3.86E-12 |
| 196 | ENSMUSG00000028957 | *PER3* | 1137.75 | 4664.75 | 1.78 | 2.36E-12 |
| 197 | ENSMUSG00000037904 | *ANKRD9* | 178 | 1107.75 | 2.41 | 1.74E-12 |
| 198 | ENSMUSG00000035376 | *HACD2* | 5003.5 | 14507.75 | 1.37 | 1.70E-12 |
| 199 | ENSMUSG00000110496 | *Gm45909* | 577.25 | 2416.5 | 1.92 | 1.46E-12 |
| 200 | ENSMUSG00000110613 | *Lncbate1* | 899.25 | 3417 | 1.67 | 1.11E-12 |
| 201 | ENSMUSG00000111840 | *Gm48832* | 609 | 2624.5 | 2.00 | 8.97E-13 |
| 202 | ENSMUSG00000020572 | *NAMPT* | 3837.25 | 11378.25 | 1.42 | 6.15E-13 |
| 203 | ENSMUSG00000086765 | *GM11827* | 29.5 | 241.5 | 2.75 | 4.31E-13 |
| 204 | ENSMUSG00000113175 | *Gm9973* | 16.25 | 126.25 | 2.79 | 3.83E-13 |
| 205 | ENSMUSG00000055320 | *TEAD1* | 1820.25 | 8929.5 | 2.16 | 1.66E-13 |
| 206 | ENSMUSG00000113491 | *Gm19221* | 65.5 | 425 | 2.54 | 1.37E-13 |
| 207 | ENSMUSG00000026473 | *GLUL* | 37839.5 | 121416.5 | 1.43 | 7.16E-14 |
| 208 | ENSMUSG00000113898 | *Gm19144* | 318.25 | 1875 | 2.44 | 1.82E-14 |
| 209 | ENSMUSG00000024222 | *FKBP5* | 1072.5 | 8854.75 | 2.72 | 1.63E-14 |
| 210 | ENSMUSG00000032010 | *USP2* | 350 | 1811.25 | 2.11 | 1.09E-14 |
| 211 | ENSMUSG00000022389 | *TEF* | 3434.5 | 13913 | 1.74 | 6.13E-15 |
| 212 | ENSMUSG00000021750 | *FAM107A* | 93.5 | 658.25 | 2.57 | 5.23E-15 |
| 213 | ENSMUSG00000115801 | *AC160336.1* | 646.25 | 3335.75 | 2.08 | 3.49E-16 |
| 214 | ENSMUSG00000038550 | *CIART* | 52.5 | 549.75 | 3.11 | 1.41E-16 |
| 215 | ENSMUSG00000028862 | *MAP3K6* | 861.75 | 4019 | 2.02 | 4.37E-18 |
| 216 | ENSMUSG00000041020 | *MAP7D2* | 5.75 | 151.75 | 4.47 | 6.61E-20 |
| 217 | ENSMUSG00000028207 | *ASPH* | 2809.75 | 11166 | 1.79 | 4.36E-20 |
| 218 | ENSMUSG00000095061 | *E030018B13RIK* | 5.5 | 133 | 4.40 | 1.80E-20 |
| 219 | ENSMUSG00000042540 | *ACOT5* | 6.5 | 149 | 4.42 | 6.39E-21 |
| 220 | ENSMUSG00000028655 | *MFSD2A* | 19.75 | 459 | 4.22 | 3.57E-23 |
| 221 | ENSMUSG00000055866 | *PER2* | 280 | 2896 | 3.15 | 4.21E-26 |

Table S3 Downregulated genes (*p*<0.05 and log2FC >1)

| No. | Gene_ID | Gene Name | WT(FPKM) | KI(FPKM) | log2FC | *p*_value |
| --- | --- | --- | --- | --- | --- | --- |
| 1 | ENSMUSG00000022491 | *Glycam1* | 247.25 | 3 | -6.90 | 1.56E-03 |
| 2 | ENSMUSG00000059898 | *Dsc3* | 119.75 | 3 | -5.80 | 4.74E-03 |
| 3 | ENSMUSG00000021490 | *Slc34a1* | 183.5 | 6.5 | -5.55 | 1.04E-02 |
| 4 | ENSMUSG00000024673 | *MS4A1* | 710.25 | 20 | -5.42 | 2.11E-04 |
| 5 | ENSMUSG00000022416 | *CACNA1I* | 195 | 5.75 | -5.42 | 3.01E-04 |
| 6 | ENSMUSG00000028307 | *ALDOB* | 103.5 | 3 | -5.41 | 1.16E-03 |
| 7 | ENSMUSG00000044309 | *APOL7C* | 442.25 | 14.75 | -5.34 | 6.22E-05 |
| 8 | ENSMUSG00000068105 | *TNFRSF13C* | 243.25 | 7.75 | -5.34 | 2.43E-03 |
| 9 | ENSMUSG00000005540 | *FCER2A* | 948.75 | 31 | -5.29 | 3.43E-04 |
| 10 | ENSMUSG00000014030 | *Pax5* | 796 | 26.25 | -5.26 | 3.60E-04 |
| 11 | ENSMUSG00000034634 | *Ly6d* | 321.5 | 12.25 | -5.14 | 2.92E-04 |
| 12 | ENSMUSG00000008193 | *SPIB* | 592.75 | 22.75 | -5.08 | 4.93E-04 |
| 13 | ENSMUSG00000030724 | *CD19* | 1256.75 | 46 | -5.08 | 4.93E-04 |
| 14 | ENSMUSG00000024334 | *H2-OA* | 134.25 | 4.5 | -5.07 | 1.14E-04 |
| 15 | ENSMUSG00000003379 | *CD79A* | 1479.25 | 60.5 | -5.02 | 4.53E-04 |
| 16 | ENSMUSG00000042474 | *FCMR* | 714.25 | 31 | -5.01 | 1.14E-03 |
| 17 | ENSMUSG00000040899 | *CCR6* | 97 | 3.25 | -4.99 | 1.42E-03 |
| 18 | ENSMUSG00000104213 | *Ighd* | 2355.75 | 96.25 | -4.93 | 2.09E-04 |
| 19 | ENSMUSG00000047880 | *CXCR5* | 242.25 | 9.75 | -4.90 | 1.64E-04 |
| 20 | ENSMUSG00000032758 | *Kap* | 155 | 6.5 | -4.85 | 1.93E-02 |
| 21 | ENSMUSG00000040592 | *CD79B* | 878.75 | 37.25 | -4.83 | 3.04E-04 |
| 22 | ENSMUSG00000071068 | *TREML2* | 203.75 | 9.25 | -4.76 | 7.56E-05 |
| 23 | ENSMUSG00000026616 | *CR2* | 441 | 21.5 | -4.75 | 1.21E-03 |
| 24 | ENSMUSG00000059994 | *FCRL1* | 281.25 | 13 | -4.72 | 1.35E-03 |
| 25 | ENSMUSG00000032053 | *POU2AF1* | 335.25 | 15.25 | -4.72 | 9.18E-04 |
| 26 | ENSMUSG00000030577 | *CD22* | 1077 | 54.75 | -4.65 | 1.14E-03 |
| 27 | ENSMUSG00000067341 | *H2-Eb2* | 139.25 | 7.25 | -4.61 | 1.07E-03 |
| 28 | ENSMUSG00000029603 | *DTX1* | 205 | 10.25 | -4.51 | 2.54E-04 |
| 29 | ENSMUSG00000030468 | *SIGLECG* | 599.75 | 35 | -4.47 | 3.31E-04 |
| 30 | ENSMUSG00000014453 | *BLK* | 292.25 | 16.75 | -4.46 | 1.48E-03 |
| 31 | ENSMUSG00000026390 | *MARCO* | 314 | 18.5 | -4.46 | 4.53E-04 |
| 32 | ENSMUSG00000039760 | *IL22RA2* | 176.5 | 10.25 | -4.40 | 4.28E-04 |
| 33 | ENSMUSG00000000861 | *BCL11A* | 195.25 | 13.5 | -4.23 | 5.70E-04 |
| 34 | ENSMUSG00000024399 | *LTB* | 641 | 43.5 | -4.22 | 1.57E-04 |
| 35 | ENSMUSG00000094686 | *Ccl21a* | 641.25 | 47 | -4.13 | 2.05E-04 |
| 36 | ENSMUSG00000041538 | *H2-OB* | 899.75 | 64 | -4.12 | 4.23E-04 |
| 37 | ENSMUSG00000037944 | *CCR7* | 329.25 | 24.25 | -4.10 | 3.43E-04 |
| 38 | ENSMUSG00000076937 | *Iglc2* | 206 | 14 | -4.05 | 7.16E-03 |
| 39 | ENSMUSG00000024675 | *Ms4a4c* | 244.5 | 18.5 | -4.05 | 8.85E-06 |
| 40 | ENSMUSG00000037548 | *H2-DMB2* | 633 | 49 | -4.00 | 1.37E-04 |
| 41 | ENSMUSG00000031779 | *CCL22* | 232.25 | 19.25 | -3.99 | 1.27E-03 |
| 42 | ENSMUSG00000038421 | *Fcrla* | 184 | 14.25 | -3.96 | 1.02E-03 |
| 43 | ENSMUSG00000026077 | *NPAS2* | 210 | 18 | -3.76 | 2.32E-14 |
| 44 | ENSMUSG00000074071 | *FAM169B* | 122 | 11.25 | -3.75 | 3.62E-03 |
| 45 | ENSMUSG00000022696 | *SIDT1* | 128.25 | 11.25 | -3.74 | 1.84E-03 |
| 46 | ENSMUSG00000030745 | *Il21r* | 434.25 | 44 | -3.72 | 1.04E-03 |
| 47 | ENSMUSG00000113136 | *AC133083.2* | 147 | 13.5 | -3.70 | 1.86E-03 |
| 48 | ENSMUSG00000026581 | *SELL* | 1075.25 | 101.25 | -3.69 | 1.11E-03 |
| 49 | ENSMUSG00000057337 | *CHST3* | 579.25 | 57.5 | -3.68 | 1.13E-03 |
| 50 | ENSMUSG00000024669 | *CD5* | 158.25 | 14.5 | -3.58 | 2.70E-03 |
| 51 | ENSMUSG00000052013 | *BTLA* | 641.5 | 65.75 | -3.53 | 2.55E-03 |
| 52 | ENSMUSG00000018168 | *IKZF3* | 467.5 | 52.75 | -3.42 | 2.54E-03 |
| 53 | ENSMUSG00000050600 | *ZFP831* | 170.25 | 19 | -3.41 | 3.26E-03 |
| 54 | ENSMUSG00000037922 | *BANK1* | 391.5 | 47.25 | -3.38 | 2.94E-05 |
| 55 | ENSMUSG00000027985 | *LEF1* | 166.25 | 18.5 | -3.34 | 1.49E-03 |
| 56 | ENSMUSG00000000782 | *Tcf7* | 642.25 | 73.5 | -3.34 | 1.43E-03 |
| 57 | ENSMUSG00000051212 | *GPR183* | 159 | 21.25 | -3.30 | 1.08E-04 |
| 58 | ENSMUSG00000044770 | *SCML4* | 148 | 17.75 | -3.29 | 1.06E-03 |
| 59 | ENSMUSG00000076609 | *Igkc* | 1400 | 186.5 | -3.26 | 1.70E-03 |
| 60 | ENSMUSG00000020297 | *NSG2* | 123.75 | 14.5 | -3.26 | 5.23E-03 |
| 61 | ENSMUSG00000045826 | *PTPRCAP* | 430.25 | 58 | -3.20 | 1.03E-03 |
| 62 | ENSMUSG00000015314 | *Slamf6* | 175 | 21.75 | -3.19 | 4.41E-03 |
| 63 | ENSMUSG00000039981 | *ZC3H12D* | 136.75 | 19.5 | -3.14 | 2.31E-04 |
| 64 | ENSMUSG00000033730 | *EGR3* | 242 | 35.5 | -3.12 | 1.22E-06 |
| 65 | ENSMUSG00000022686 | *B3GNT5* | 245.75 | 36.75 | -3.04 | 2.61E-03 |
| 66 | ENSMUSG00000063646 | *JAKMIP1* | 93 | 13.5 | -3.01 | 1.61E-03 |
| 67 | ENSMUSG00000085887 | *ARHGAP27OS3* | 103.75 | 15 | -3.01 | 8.13E-03 |
| 68 | ENSMUSG00000076928 | *Trac* | 296.5 | 43.75 | -3.01 | 3.22E-03 |
| 69 | ENSMUSG00000037849 | *Ifi206* | 264.75 | 42 | -3.00 | 4.79E-05 |
| 70 | ENSMUSG00000076498 | *Trbc2* | 364.25 | 54.75 | -3.00 | 1.90E-03 |
| 71 | ENSMUSG00000053044 | *CD8B1* | 174.5 | 29 | -3.00 | 8.97E-03 |
| 72 | ENSMUSG00000042351 | *Grap2* | 277.25 | 39.5 | -3.00 | 3.96E-03 |
| 73 | ENSMUSG00000064065 | *IPCEF1* | 135.25 | 19 | -2.99 | 1.34E-03 |
| 74 | ENSMUSG00000048251 | *BCL11B* | 171.25 | 25.5 | -2.99 | 4.94E-03 |
| 75 | ENSMUSG00000036526 | *CARD11* | 276.5 | 47 | -2.95 | 3.96E-03 |
| 76 | ENSMUSG00000024670 | *CD6* | 169.75 | 26.5 | -2.94 | 5.47E-03 |
| 77 | ENSMUSG00000023274 | *CD4* | 310.75 | 48 | -2.93 | 2.02E-03 |
| 78 | ENSMUSG00000002033 | *CD3G* | 208.25 | 32.25 | -2.86 | 3.24E-03 |
| 79 | ENSMUSG00000052142 | *RASAL3* | 766.25 | 134.75 | -2.84 | 2.64E-03 |
| 80 | ENSMUSG00000001588 | *Acap1* | 763.25 | 132.25 | -2.81 | 3.00E-03 |
| 81 | ENSMUSG00000032094 | *CD3D* | 135.5 | 23.25 | -2.80 | 2.54E-03 |
| 82 | ENSMUSG00000010142 | *Tnfrsf13b* | 276.25 | 52.25 | -2.79 | 4.49E-04 |
| 83 | ENSMUSG00000040247 | *Tbc1d10c* | 442.75 | 81.75 | -2.79 | 1.98E-03 |
| 84 | ENSMUSG00000052749 | *TRIM30B* | 140.5 | 27.5 | -2.78 | 5.12E-03 |
| 85 | ENSMUSG00000032093 | *CD3E* | 237 | 41.5 | -2.77 | 4.29E-03 |
| 86 | ENSMUSG00000025279 | *Dnase1l3* | 326 | 56.5 | -2.76 | 1.31E-03 |
| 87 | ENSMUSG00000040350 | *TRIM7* | 230.25 | 46.5 | -2.76 | 4.64E-04 |
| 88 | ENSMUSG00000073008 | *GPR174* | 134.5 | 23.5 | -2.75 | 9.46E-03 |
| 89 | ENSMUSG00000070691 | *RUNX3* | 141.5 | 25.75 | -2.74 | 4.19E-03 |
| 90 | ENSMUSG00000037318 | *TRAF3IP3* | 370 | 68.5 | -2.71 | 4.28E-03 |
| 91 | ENSMUSG00000053977 | *CD8A* | 325 | 61.5 | -2.71 | 1.02E-02 |
| 92 | ENSMUSG00000022504 | *CIITA* | 710.5 | 139.5 | -2.71 | 1.02E-04 |
| 93 | ENSMUSG00000000409 | *Lck* | 467.75 | 87.5 | -2.71 | 3.87E-03 |
| 94 | ENSMUSG00000097636 | *MIRT1* | 117.5 | 21.25 | -2.71 | 2.69E-03 |
| 95 | ENSMUSG00000005465 | *IL27RA* | 330 | 60.75 | -2.71 | 6.63E-04 |
| 96 | ENSMUSG00000027368 | *DUSP2* | 139.5 | 26.5 | -2.71 | 4.77E-03 |
| 97 | ENSMUSG00000028459 | *CD72* | 398.25 | 77.75 | -2.69 | 1.68E-03 |
| 98 | ENSMUSG00000020340 | *CYFIP2* | 497 | 96.25 | -2.69 | 2.35E-03 |
| 99 | ENSMUSG00000029204 | *RHOH* | 179 | 34.25 | -2.68 | 2.49E-03 |
| 100 | ENSMUSG00000026594 | *Ralgps2* | 515.25 | 103.25 | -2.66 | 8.77E-04 |
| 101 | ENSMUSG00000030742 | *LAT* | 283 | 52.25 | -2.65 | 5.40E-03 |
| 102 | ENSMUSG00000002204 | *NAPSA* | 223 | 47.5 | -2.64 | 3.21E-03 |
| 103 | ENSMUSG00000008496 | *Pou2f2* | 394 | 87.5 | -2.58 | 8.03E-04 |
| 104 | ENSMUSG00000026979 | *PSD4* | 474 | 102.5 | -2.55 | 8.55E-04 |
| 105 | ENSMUSG00000035095 | *FAM167A* | 130.75 | 28.25 | -2.54 | 9.87E-07 |
| 106 | ENSMUSG00000073902 | *GM1966* | 243.5 | 54 | -2.53 | 6.88E-04 |
| 107 | ENSMUSG00000051998 | *LAX1* | 260.25 | 56.75 | -2.51 | 4.49E-03 |
| 108 | ENSMUSG00000074497 | *A430078G23RIK* | 140.5 | 29.25 | -2.51 | 5.76E-03 |
| 109 | ENSMUSG00000084796 | *Mir142hg* | 106.25 | 25 | -2.51 | 6.27E-03 |
| 110 | ENSMUSG00000004266 | *PTPN6* | 1012.25 | 238 | -2.50 | 6.78E-04 |
| 111 | ENSMUSG00000056145 | *AI504432* | 102.75 | 22.5 | -2.49 | 4.97E-03 |
| 112 | ENSMUSG00000006731 | *B4GALNT1* | 581.75 | 132.25 | -2.49 | 1.79E-03 |
| 113 | ENSMUSG00000044827 | *TLR1* | 289.5 | 68.25 | -2.49 | 2.16E-03 |
| 114 | ENSMUSG00000020395 | *ITK* | 212.25 | 47.25 | -2.48 | 1.65E-02 |
| 115 | ENSMUSG00000066677 | *IFI208* | 242.5 | 56.25 | -2.48 | 2.21E-04 |
| 116 | ENSMUSG00000036006 | *RIPOR2* | 550.25 | 128 | -2.47 | 2.16E-03 |
| 117 | ENSMUSG00000036362 | *P2RY13* | 94.25 | 22.75 | -2.47 | 4.33E-04 |
| 118 | ENSMUSG00000050357 | *CARMIL2* | 321.75 | 70.5 | -2.46 | 4.69E-03 |
| 119 | ENSMUSG00000098112 | *BIN2* | 324.5 | 77 | -2.46 | 1.73E-03 |
| 120 | ENSMUSG00000030336 | *CD27* | 187.75 | 41.25 | -2.45 | 8.51E-03 |
| 121 | ENSMUSG00000050921 | *P2RY10* | 185.75 | 41.75 | -2.42 | 7.72E-03 |
| 122 | ENSMUSG00000000682 | *Cd52* | 766 | 188 | -2.40 | 8.01E-04 |
| 123 | ENSMUSG00000027347 | *RASGRP1* | 279.5 | 64.25 | -2.40 | 6.97E-03 |
| 124 | ENSMUSG00000017639 | *RAB11FIP4* | 192.75 | 36.25 | -2.40 | 2.55E-06 |
| 125 | ENSMUSG00000023169 | *SLC38A1* | 340.5 | 82.75 | -2.39 | 3.11E-03 |
| 126 | ENSMUSG00000038352 | *Arl5c* | 157.5 | 38 | -2.37 | 2.79E-03 |
| 127 | ENSMUSG00000035606 | *KY* | 133.75 | 35.5 | -2.37 | 9.85E-03 |
| 128 | ENSMUSG00000028874 | *Fgr* | 151.25 | 37.25 | -2.35 | 3.61E-04 |
| 129 | ENSMUSG00000026009 | *ICOS* | 113.75 | 26.75 | -2.35 | 5.75E-03 |
| 130 | ENSMUSG00000030091 | *NUP210* | 635.25 | 155.25 | -2.34 | 2.24E-03 |
| 131 | ENSMUSG00000043263 | *IFI209* | 709 | 184.75 | -2.34 | 7.81E-04 |
| 132 | ENSMUSG00000030830 | *ITGAL* | 619.75 | 150.75 | -2.32 | 1.79E-03 |
| 133 | ENSMUSG00000073491 | *IFI213* | 506.75 | 130.5 | -2.32 | 6.93E-04 |
| 134 | ENSMUSG00000029254 | *STAP1* | 211 | 51.5 | -2.32 | 3.75E-03 |
| 135 | ENSMUSG00000061132 | *BLNK* | 300.25 | 81.25 | -2.31 | 8.33E-04 |
| 136 | ENSMUSG00000027863 | *CD2* | 263 | 64.5 | -2.31 | 5.10E-03 |
| 137 | ENSMUSG00000047415 | *GPR68* | 90 | 23 | -2.31 | 1.47E-03 |
| 138 | ENSMUSG00000031101 | *Sash3* | 563.25 | 144 | -2.30 | 8.30E-04 |
| 139 | ENSMUSG00000026980 | *LY75* | 105 | 25.25 | -2.29 | 2.61E-03 |
| 140 | ENSMUSG00000026832 | *CYTIP* | 530.5 | 132.75 | -2.27 | 1.22E-03 |
| 141 | ENSMUSG00000000673 | *Haao* | 152.25 | 40.5 | -2.27 | 1.09E-02 |
| 142 | ENSMUSG00000023947 | *NFKBIE* | 252.25 | 66 | -2.26 | 1.22E-04 |
| 143 | ENSMUSG00000100658 | *F730311O21RIK* | 155.25 | 43.5 | -2.26 | 2.73E-04 |
| 144 | ENSMUSG00000021298 | *GPR132* | 293.5 | 79 | -2.22 | 2.07E-03 |
| 145 | ENSMUSG00000057058 | *Skap1* | 99.5 | 24.5 | -2.22 | 1.69E-02 |
| 146 | ENSMUSG00000044938 | *KLHL31* | 165 | 46 | -2.22 | 1.80E-02 |
| 147 | ENSMUSG00000030798 | *CD37* | 1688.25 | 480.25 | -2.20 | 2.23E-03 |
| 148 | ENSMUSG00000026117 | *ZAP70* | 149.5 | 39 | -2.20 | 9.51E-03 |
| 149 | ENSMUSG00000040061 | *PLCB2* | 288.75 | 76 | -2.19 | 2.79E-03 |
| 150 | ENSMUSG00000026875 | *TRAF1* | 399.25 | 107.25 | -2.19 | 4.11E-03 |
| 151 | ENSMUSG00000075033 | *NXPE3* | 134 | 36.25 | -2.19 | 9.45E-03 |
| 152 | ENSMUSG00000070000 | *Fcho1* | 280.75 | 80 | -2.18 | 1.47E-02 |
| 153 | ENSMUSG00000057948 | *UNC13D* | 241.25 | 69.5 | -2.18 | 4.85E-03 |
| 154 | ENSMUSG00000030707 | *CORO1A* | 2368.5 | 673 | -2.17 | 1.12E-03 |
| 155 | ENSMUSG00000079563 | *Pglyrp2* | 86 | 24.25 | -2.16 | 3.23E-02 |
| 156 | ENSMUSG00000062585 | *CNR2* | 169.75 | 48.25 | -2.16 | 9.99E-03 |
| 157 | ENSMUSG00000039747 | *Orai2* | 332.25 | 91 | -2.16 | 1.42E-03 |
| 158 | ENSMUSG00000061577 | *ADGRG5* | 96 | 27.25 | -2.15 | 1.24E-02 |
| 159 | ENSMUSG00000076490 | *Trbc1* | 198 | 51.75 | -2.15 | 1.15E-02 |
| 160 | ENSMUSG00000033220 | *RAC2* | 1472.75 | 429.25 | -2.14 | 1.55E-03 |
| 161 | ENSMUSG00000026100 | *MSTN* | 117 | 23.25 | -2.13 | 1.26E-02 |
| 162 | ENSMUSG00000022876 | *SAMSN1* | 174 | 51.75 | -2.13 | 7.85E-03 |
| 163 | ENSMUSG00000055116 | *ARNTL* | 380 | 95 | -2.12 | 1.12E-10 |
| 164 | ENSMUSG00000045322 | *TLR9* | 182.5 | 54.75 | -2.11 | 2.31E-03 |
| 165 | ENSMUSG00000037280 | *Galnt6* | 194 | 58.75 | -2.10 | 1.32E-02 |
| 166 | ENSMUSG00000021624 | *CD180* | 281.75 | 86 | -2.10 | 7.74E-04 |
| 167 | ENSMUSG00000030365 | *CLEC2I* | 264.5 | 76.5 | -2.10 | 4.02E-03 |
| 168 | ENSMUSG00000051457 | *SPN* | 371.75 | 103.25 | -2.09 | 2.38E-03 |
| 169 | ENSMUSG00000026012 | *CD28* | 106.75 | 29.5 | -2.09 | 1.21E-02 |
| 170 | ENSMUSG00000021451 | *SEMA4D* | 425 | 126.25 | -2.08 | 2.92E-03 |
| 171 | ENSMUSG00000003352 | *CACNB3* | 386.25 | 119.75 | -2.07 | 6.32E-03 |
| 172 | ENSMUSG00000090066 | *1110002E22Rik* | 136.5 | 42.75 | -2.07 | 1.29E-02 |
| 173 | ENSMUSG00000050592 | *FAM78A* | 356.25 | 107.25 | -2.07 | 2.34E-03 |
| 174 | ENSMUSG00000037946 | *FGD3* | 258.75 | 77.25 | -2.07 | 8.99E-04 |
| 175 | ENSMUSG00000056290 | *MS4A4B* | 391.75 | 108 | -2.06 | 8.89E-03 |
| 176 | ENSMUSG00000041642 | *KIF21B* | 934.25 | 283.5 | -2.05 | 4.46E-03 |
| 177 | ENSMUSG00000058470 | *GM8369* | 128.5 | 39.75 | -2.05 | 3.66E-02 |
| 178 | ENSMUSG00000034959 | *RUBCNL* | 152.25 | 47.75 | -2.04 | 1.08E-02 |
| 179 | ENSMUSG00000039264 | *GIMAP3* | 1202.5 | 351 | -2.04 | 9.24E-03 |
| 180 | ENSMUSG00000033450 | *TAGAP* | 95 | 27.75 | -2.04 | 3.16E-03 |
| 181 | ENSMUSG00000051506 | *WDFY4* | 725.75 | 223.25 | -2.03 | 2.39E-03 |
| 182 | ENSMUSG00000003882 | *IL7R* | 252.25 | 71 | -2.03 | 2.16E-02 |
| 183 | ENSMUSG00000064267 | *HVCN1* | 496 | 141.25 | -2.02 | 4.11E-05 |
| 184 | ENSMUSG00000054065 | *PKP3* | 90.5 | 27.5 | -2.01 | 2.49E-03 |
| 185 | ENSMUSG00000031389 | *Arhgap4* | 747.25 | 239.75 | -2.00 | 4.39E-03 |
| 186 | ENSMUSG00000026395 | *PTPRC* | 2458.25 | 762.75 | -2.00 | 1.83E-03 |
| 187 | ENSMUSG00000074785 | *PLXNC1* | 304.75 | 99.5 | -1.99 | 1.24E-03 |
| 188 | ENSMUSG00000052889 | *PRKCB* | 416.75 | 132.5 | -1.99 | 8.34E-04 |
| 189 | ENSMUSG00000000486 | *SEPT1* | 590 | 177.25 | -1.98 | 3.72E-03 |
| 190 | ENSMUSG00000021880 | *Rnase6* | 189.5 | 60.25 | -1.98 | 3.37E-03 |
| 191 | ENSMUSG00000036931 | *NFKBID* | 140.5 | 45.75 | -1.97 | 1.23E-02 |
| 192 | ENSMUSG00000002007 | *SRPK3* | 192.25 | 62.25 | -1.97 | 1.88E-02 |
| 193 | ENSMUSG00000025017 | *Pik3ap1* | 316.25 | 106.5 | -1.97 | 2.58E-04 |
| 194 | ENSMUSG00000027470 | *MYLK2* | 357.5 | 119.5 | -1.96 | 4.10E-02 |
| 195 | ENSMUSG00000017652 | *Cd40* | 175.75 | 60 | -1.94 | 4.91E-03 |
| 196 | ENSMUSG00000026070 | *IL18R1* | 173 | 58.75 | -1.93 | 7.33E-05 |
| 197 | ENSMUSG00000047945 | *MARCKSL1* | 563.5 | 186.5 | -1.92 | 8.71E-04 |
| 198 | ENSMUSG00000022602 | *ARC* | 105.75 | 31.75 | -1.92 | 9.27E-04 |
| 199 | ENSMUSG00000000244 | *TSPAN32* | 311.5 | 107 | -1.91 | 3.00E-03 |
| 200 | ENSMUSG00000041481 | *Serpina3g* | 254 | 87 | -1.91 | 1.78E-02 |
| 201 | ENSMUSG00000037370 | *ENPP1* | 87.75 | 29.75 | -1.91 | 3.25E-03 |
| 202 | ENSMUSG00000040829 | *ZMYND15* | 155.75 | 52.5 | -1.90 | 2.88E-03 |
| 203 | ENSMUSG00000003283 | *HCK* | 147.5 | 53.5 | -1.90 | 5.39E-03 |
| 204 | ENSMUSG00000037337 | *MAP4K1* | 424.5 | 146 | -1.89 | 6.06E-03 |
| 205 | ENSMUSG00000035042 | *Ccl5* | 429.25 | 142.25 | -1.87 | 1.22E-02 |
| 206 | ENSMUSG00000040270 | *BACH2* | 331.75 | 114 | -1.86 | 1.03E-02 |
| 207 | ENSMUSG00000051504 | *Siglech* | 111 | 36.75 | -1.86 | 3.62E-04 |
| 208 | ENSMUSG00000025203 | *SCD2* | 20402.75 | 5925.75 | -1.86 | 1.33E-08 |
| 209 | ENSMUSG00000055546 | *TIMD4* | 318.75 | 112.75 | -1.86 | 2.22E-03 |
| 210 | ENSMUSG00000078763 | *SLFN1* | 195 | 68.5 | -1.86 | 5.14E-03 |
| 211 | ENSMUSG00000040663 | *CLCF1* | 357.75 | 118.25 | -1.86 | 1.26E-02 |
| 212 | ENSMUSG00000039395 | *MREG* | 109 | 38.5 | -1.86 | 1.14E-02 |
| 213 | ENSMUSG00000015312 | *GADD45B* | 224.75 | 73.25 | -1.86 | 3.37E-04 |
| 214 | ENSMUSG00000049866 | *ARL4C* | 488.25 | 161 | -1.86 | 3.40E-03 |
| 215 | ENSMUSG00000034330 | *PLCG2* | 430.5 | 153.5 | -1.85 | 3.19E-03 |
| 216 | ENSMUSG00000039783 | *Kmo* | 223.25 | 80 | -1.85 | 1.28E-03 |
| 217 | ENSMUSG00000042817 | *FLT3* | 99.5 | 34.25 | -1.84 | 1.91E-02 |
| 218 | ENSMUSG00000054342 | *KCNN4* | 174.5 | 61.5 | -1.84 | 2.62E-03 |
| 219 | ENSMUSG00000023034 | *NR4A1* | 1597.5 | 512.25 | -1.84 | 8.02E-04 |
| 220 | ENSMUSG00000039158 | *Akna* | 994 | 353.5 | -1.84 | 2.80E-03 |
| 221 | ENSMUSG00000039774 | *GALNT12* | 88.25 | 30.5 | -1.84 | 4.19E-03 |
| 222 | ENSMUSG00000021998 | *Lcp1* | 3073.5 | 1100.25 | -1.83 | 1.31E-04 |
| 223 | ENSMUSG00000046818 | *DDIT4L* | 90.75 | 31 | -1.82 | 5.89E-03 |
| 224 | ENSMUSG00000035697 | *ARHGAP45* | 2829.75 | 1021.5 | -1.81 | 3.54E-03 |
| 225 | ENSMUSG00000028071 | *Sh2d2a* | 188.5 | 66.25 | -1.81 | 3.82E-02 |
| 226 | ENSMUSG00000049871 | *NLRC3* | 164.75 | 57 | -1.80 | 1.98E-02 |
| 227 | ENSMUSG00000034171 | *FAAH* | 144.75 | 50.5 | -1.80 | 2.05E-02 |
| 228 | ENSMUSG00000026778 | *PRKCQ* | 234 | 85 | -1.80 | 3.39E-03 |
| 229 | ENSMUSG00000038179 | *SLAMF7* | 191 | 70 | -1.79 | 3.17E-03 |
| 230 | ENSMUSG00000015396 | *Cd83* | 435.25 | 150.5 | -1.79 | 1.05E-04 |
| 231 | ENSMUSG00000042717 | *PPP1R3A* | 100.5 | 36.25 | -1.79 | 3.21E-02 |
| 232 | ENSMUSG00000044734 | *SERPINB1A* | 317 | 118 | -1.79 | 1.23E-03 |
| 233 | ENSMUSG00000037379 | *Spon2* | 355.75 | 115.25 | -1.79 | 5.87E-07 |
| 234 | ENSMUSG00000004707 | *LY9* | 279.5 | 111 | -1.79 | 1.22E-02 |
| 235 | ENSMUSG00000039982 | *DTX4* | 1756.25 | 554.75 | -1.78 | 7.68E-09 |
| 236 | ENSMUSG00000018654 | *IKZF1* | 850.5 | 309.75 | -1.77 | 3.62E-03 |
| 237 | ENSMUSG00000043243 | *FAM129C* | 106.5 | 37.75 | -1.76 | 5.88E-03 |
| 238 | ENSMUSG00000022014 | *Epsti1* | 706 | 264 | -1.76 | 3.14E-03 |
| 239 | ENSMUSG00000038418 | *Egr1* | 1029.75 | 324.25 | -1.75 | 3.47E-05 |
| 240 | ENSMUSG00000020437 | *MYO1G* | 974 | 373.75 | -1.75 | 5.94E-03 |
| 241 | ENSMUSG00000016756 | *CMAH* | 889.5 | 338.25 | -1.75 | 2.13E-04 |
| 242 | ENSMUSG00000003070 | *EFNA2* | 92.75 | 33 | -1.74 | 3.09E-06 |
| 243 | ENSMUSG00000031506 | *PTPN7* | 242.25 | 89.25 | -1.74 | 1.99E-03 |
| 244 | ENSMUSG00000035711 | *Dok3* | 531.5 | 209 | -1.72 | 1.93E-02 |
| 245 | ENSMUSG00000021200 | *ASB2* | 171.75 | 67.75 | -1.72 | 5.63E-03 |
| 246 | ENSMUSG00000023078 | *CXCL13* | 499.5 | 204.25 | -1.71 | 1.97E-02 |
| 247 | ENSMUSG00000071715 | *NCF4* | 291.75 | 115 | -1.71 | 6.21E-03 |
| 248 | ENSMUSG00000056708 | *IER5* | 596.25 | 216.5 | -1.70 | 1.42E-04 |
| 249 | ENSMUSG00000031906 | *SMPD3* | 1029.25 | 437.5 | -1.70 | 7.49E-04 |
| 250 | ENSMUSG00000028278 | *RRAGD* | 102.75 | 40 | -1.68 | 4.88E-03 |
| 251 | ENSMUSG00000022044 | *STMN4* | 82 | 33.75 | -1.68 | 8.74E-04 |
| 252 | ENSMUSG00000041202 | *PLA2G2D* | 451 | 166 | -1.68 | 2.82E-04 |
| 253 | ENSMUSG00000048865 | *ARHGAP30* | 1302 | 518.75 | -1.68 | 2.90E-03 |
| 254 | ENSMUSG00000040699 | *LIMD2* | 1260.25 | 487 | -1.68 | 1.73E-03 |
| 255 | ENSMUSG00000045165 | *AI467606* | 322 | 130.5 | -1.67 | 2.51E-03 |
| 256 | ENSMUSG00000041515 | *IRF8* | 916.75 | 361.5 | -1.67 | 2.75E-03 |
| 257 | ENSMUSG00000052397 | *EZR* | 643.25 | 242.25 | -1.67 | 1.05E-02 |
| 258 | ENSMUSG00000031304 | *IL2RG* | 518.75 | 198.25 | -1.67 | 7.15E-03 |
| 259 | ENSMUSG00000032661 | *OAS3* | 183.5 | 77 | -1.67 | 9.65E-03 |
| 260 | ENSMUSG00000027230 | *Creb3l1* | 909.5 | 349.5 | -1.66 | 1.10E-08 |
| 261 | ENSMUSG00000034881 | *TBXA2R* | 87.5 | 32.25 | -1.66 | 2.28E-03 |
| 262 | ENSMUSG00000031264 | *BTK* | 298.25 | 123.25 | -1.65 | 9.28E-03 |
| 263 | ENSMUSG00000000120 | *NGFR* | 94.75 | 37 | -1.65 | 1.09E-04 |
| 264 | ENSMUSG00000029299 | *ABCG3* | 180.5 | 73.25 | -1.65 | 5.67E-03 |
| 265 | ENSMUSG00000074472 | *ZFP872* | 102.5 | 37 | -1.65 | 8.30E-06 |
| 266 | ENSMUSG00000052760 | *A630001G21Rik* | 363.75 | 139.75 | -1.65 | 1.40E-02 |
| 267 | ENSMUSG00000003545 | *FOSB* | 162.5 | 49 | -1.62 | 1.64E-02 |
| 268 | ENSMUSG00000043931 | *GIMAP7* | 88.5 | 35.75 | -1.61 | 2.93E-02 |
| 269 | ENSMUSG00000034438 | *Gbp8* | 136.25 | 55 | -1.60 | 2.28E-02 |
| 270 | ENSMUSG00000039891 | *TXLNB* | 196.25 | 83.25 | -1.59 | 2.73E-02 |
| 271 | ENSMUSG00000079110 | *CAPN3* | 83 | 34.75 | -1.59 | 4.76E-03 |
| 272 | ENSMUSG00000020623 | *MAP2K6* | 174.25 | 62 | -1.58 | 1.89E-05 |
| 273 | ENSMUSG00000034317 | *TRIM59* | 140.25 | 53.75 | -1.58 | 3.52E-02 |
| 274 | ENSMUSG00000040694 | *APOBEC2* | 253 | 95 | -1.57 | 3.02E-02 |
| 275 | ENSMUSG00000054702 | *AP1S3* | 136 | 55.75 | -1.56 | 8.94E-03 |
| 276 | ENSMUSG00000001027 | *SCN4A* | 213.75 | 98.5 | -1.56 | 2.92E-02 |
| 277 | ENSMUSG00000000386 | *Mx1* | 256.5 | 112.25 | -1.55 | 2.08E-03 |
| 278 | ENSMUSG00000001281 | *ITGB7* | 691.25 | 302 | -1.55 | 1.06E-02 |
| 279 | ENSMUSG00000024617 | *CAMK2A* | 188.75 | 87.5 | -1.55 | 1.46E-02 |
| 280 | ENSMUSG00000024440 | *Pcdh12* | 391.75 | 151.5 | -1.54 | 7.34E-06 |
| 281 | ENSMUSG00000029217 | *TEC* | 195.5 | 83.5 | -1.54 | 9.64E-03 |
| 282 | ENSMUSG00000024910 | *Ctsw* | 182.75 | 73.75 | -1.53 | 3.34E-02 |
| 283 | ENSMUSG00000027009 | *ITGA4* | 543.75 | 231.75 | -1.53 | 8.16E-03 |
| 284 | ENSMUSG00000076617 | *Ighm* | 9248.75 | 3730.5 | -1.53 | 5.80E-06 |
| 285 | ENSMUSG00000020732 | *RAB37* | 83.5 | 33.75 | -1.52 | 1.30E-02 |
| 286 | ENSMUSG00000048163 | *SELPLG* | 795.75 | 359 | -1.52 | 4.57E-03 |
| 287 | ENSMUSG00000026888 | *GRB14* | 1169.25 | 377.75 | -1.51 | 5.65E-04 |
| 288 | ENSMUSG00000045763 | *BASP1* | 337.25 | 157.5 | -1.51 | 4.54E-04 |
| 289 | ENSMUSG00000020143 | *DOCK2* | 1076.75 | 487 | -1.51 | 3.85E-03 |
| 290 | ENSMUSG00000090019 | *GIMAP1* | 424.25 | 178 | -1.50 | 8.89E-03 |
| 291 | ENSMUSG00000039936 | *PIK3CD* | 1086 | 485.75 | -1.50 | 1.19E-02 |
| 292 | ENSMUSG00000031662 | *Snx20* | 177.25 | 79 | -1.50 | 1.26E-02 |
| 293 | ENSMUSG00000081189 | *Hspd1-ps4* | 492.75 | 185 | -1.49 | 4.98E-06 |
| 294 | ENSMUSG00000042842 | *SERPINB6B* | 129.5 | 55.75 | -1.48 | 4.57E-02 |
| 295 | ENSMUSG00000002983 | *Relb* | 710.5 | 295.25 | -1.47 | 1.22E-05 |
| 296 | ENSMUSG00000026430 | *Rassf5* | 523.5 | 228 | -1.47 | 9.04E-03 |
| 297 | ENSMUSG00000011008 | *MCOLN2* | 105 | 47.75 | -1.47 | 1.31E-02 |
| 298 | ENSMUSG00000023349 | *CLEC4N* | 79 | 37 | -1.46 | 1.65E-02 |
| 299 | ENSMUSG00000089876 | *TMEM102* | 97.75 | 39.25 | -1.46 | 7.48E-05 |
| 300 | ENSMUSG00000038738 | *Shank1* | 86.5 | 40 | -1.46 | 2.72E-02 |
| 301 | ENSMUSG00000003484 | *CYP4F18* | 188.75 | 91.25 | -1.45 | 2.93E-02 |
| 302 | ENSMUSG00000029075 | *Tnfrsf4* | 77.5 | 33 | -1.45 | 3.95E-02 |
| 303 | ENSMUSG00000044199 | *S1PR4* | 231.25 | 101 | -1.44 | 3.18E-02 |
| 304 | ENSMUSG00000004665 | *Cnn2* | 1538 | 672.5 | -1.44 | 4.03E-04 |
| 305 | ENSMUSG00000002668 | *Dennd1c* | 591.5 | 268.75 | -1.44 | 8.17E-03 |
| 306 | ENSMUSG00000022952 | *Runx1* | 232.25 | 100 | -1.43 | 1.24E-03 |
| 307 | ENSMUSG00000063286 | *GM8995* | 749.5 | 341.5 | -1.42 | 1.07E-02 |
| 308 | ENSMUSG00000025225 | *NFKB2* | 1092.5 | 481.25 | -1.42 | 7.32E-05 |
| 309 | ENSMUSG00000031165 | *WAS* | 367.5 | 169.5 | -1.42 | 1.12E-02 |
| 310 | ENSMUSG00000020963 | *TSHR* | 3745 | 1544.75 | -1.39 | 5.30E-08 |
| 311 | ENSMUSG00000051735 | *RINL* | 377.25 | 180 | -1.39 | 6.59E-03 |
| 312 | ENSMUSG00000031880 | *RRAD* | 146.25 | 62.5 | -1.39 | 8.27E-05 |
| 313 | ENSMUSG00000030220 | *ARHGDIB* | 1155 | 563.25 | -1.38 | 6.23E-03 |
| 314 | ENSMUSG00000033762 | *Recql4* | 130.75 | 56 | -1.38 | 2.48E-04 |
| 315 | ENSMUSG00000022508 | *BCL6* | 3452.25 | 1375 | -1.38 | 1.03E-04 |
| 316 | ENSMUSG00000109603 | *Gm32389* | 155.5 | 63.75 | -1.38 | 3.09E-04 |
| 317 | ENSMUSG00000030257 | *SRGAP3* | 144 | 66.25 | -1.38 | 4.98E-05 |
| 318 | ENSMUSG00000017817 | *JPH2* | 408.5 | 199.5 | -1.38 | 3.22E-02 |
| 319 | ENSMUSG00000040747 | *Cd53* | 1013.75 | 492 | -1.37 | 6.15E-03 |
| 320 | ENSMUSG00000089774 | *SLC5A3* | 2684.75 | 1108 | -1.37 | 5.87E-06 |
| 321 | ENSMUSG00000027843 | *PTPN22* | 221.75 | 105 | -1.36 | 2.61E-02 |
| 322 | ENSMUSG00000030047 | *ARHGAP25* | 359.25 | 169.25 | -1.36 | 5.29E-03 |
| 323 | ENSMUSG00000024737 | *SLC15A3* | 125.25 | 60.25 | -1.35 | 1.03E-03 |
| 324 | ENSMUSG00000031488 | *RAB11FIP1* | 99 | 45.75 | -1.35 | 1.20E-03 |
| 325 | ENSMUSG00000039621 | *PREX1* | 901.75 | 441.25 | -1.35 | 4.24E-03 |
| 326 | ENSMUSG00000030319 | *CAND2* | 115.25 | 55.5 | -1.35 | 1.12E-03 |
| 327 | ENSMUSG00000054400 | *CKLF* | 151.75 | 70.5 | -1.35 | 2.50E-03 |
| 328 | ENSMUSG00000059336 | *Slc14a1* | 108 | 52.75 | -1.34 | 6.17E-03 |
| 329 | ENSMUSG00000015355 | *CD48* | 305.25 | 152.75 | -1.33 | 3.69E-03 |
| 330 | ENSMUSG00000085151 | *1110018N20Rik* | 159.5 | 65.75 | -1.33 | 6.33E-04 |
| 331 | ENSMUSG00000018920 | *CXCL16* | 321.75 | 157.75 | -1.32 | 4.72E-03 |
| 332 | ENSMUSG00000057191 | *AB124611* | 216.75 | 111 | -1.32 | 2.42E-02 |
| 333 | ENSMUSG00000020120 | *PLEK* | 552.5 | 280.25 | -1.32 | 4.25E-03 |
| 334 | ENSMUSG00000040229 | *GPR34* | 128.5 | 64 | -1.32 | 8.35E-04 |
| 335 | ENSMUSG00000040345 | *ARHGAP9* | 572.5 | 291.75 | -1.32 | 1.56E-02 |
| 336 | ENSMUSG00000050106 | *TMC8* | 243 | 116.5 | -1.31 | 2.10E-02 |
| 337 | ENSMUSG00000034116 | *Vav1* | 372 | 194.25 | -1.31 | 3.60E-02 |
| 338 | ENSMUSG00000020275 | *Rel* | 393.5 | 191.5 | -1.31 | 1.17E-02 |
| 339 | ENSMUSG00000037138 | *AFF3* | 323.5 | 163.25 | -1.30 | 1.37E-02 |
| 340 | ENSMUSG00000006585 | *CDT1* | 154.5 | 74.5 | -1.30 | 1.62E-02 |
| 341 | ENSMUSG00000003348 | *MOB3A* | 192 | 93 | -1.30 | 1.10E-03 |
| 342 | ENSMUSG00000035439 | *HAUS8* | 179.5 | 87.25 | -1.30 | 2.19E-03 |
| 343 | ENSMUSG00000017737 | *MMP9* | 724.5 | 375.75 | -1.29 | 1.68E-04 |
| 344 | ENSMUSG00000030616 | *Sytl2* | 231 | 118.75 | -1.29 | 6.16E-04 |
| 345 | ENSMUSG00000038515 | *GRTP1* | 293.75 | 134 | -1.29 | 1.99E-04 |
| 346 | ENSMUSG00000072235 | *TUBA1A* | 9217.75 | 3910.25 | -1.29 | 1.32E-03 |
| 347 | ENSMUSG00000020272 | *STK10* | 840 | 410.5 | -1.29 | 4.47E-03 |
| 348 | ENSMUSG00000102752 | *GM7694* | 164.25 | 75.5 | -1.28 | 3.56E-05 |
| 349 | ENSMUSG00000053199 | *Arhgap20* | 286.25 | 143.5 | -1.28 | 1.69E-03 |
| 350 | ENSMUSG00000018983 | *E2F2* | 257.75 | 118.75 | -1.28 | 3.72E-04 |
| 351 | ENSMUSG00000089762 | *IER5L* | 185 | 86.75 | -1.28 | 3.97E-04 |
| 352 | ENSMUSG00000030263 | *Lrmp* | 527.25 | 275.5 | -1.27 | 1.61E-02 |
| 353 | ENSMUSG00000027510 | *Rbm38* | 842 | 398.25 | -1.27 | 1.24E-04 |
| 354 | ENSMUSG00000047180 | *NEURL3* | 571.5 | 292.5 | -1.27 | 5.72E-03 |
| 355 | ENSMUSG00000020882 | *Cacnb1* | 172 | 91.25 | -1.26 | 5.37E-03 |
| 356 | ENSMUSG00000047810 | *CCDC88B* | 387 | 200 | -1.26 | 1.91E-02 |
| 357 | ENSMUSG00000000732 | *ICOSL* | 737.25 | 375.75 | -1.25 | 4.24E-02 |
| 358 | ENSMUSG00000002341 | *NCAN* | 335.5 | 141.25 | -1.25 | 8.46E-03 |
| 359 | ENSMUSG00000002699 | *LCP2* | 352.75 | 184 | -1.25 | 6.82E-03 |
| 360 | ENSMUSG00000054293 | *A630033H20RIK* | 76.5 | 40 | -1.25 | 2.33E-02 |
| 361 | ENSMUSG00000028931 | *KCNAB2* | 467.25 | 257.75 | -1.25 | 1.81E-02 |
| 362 | ENSMUSG00000056418 | *BC043934* | 88.25 | 40.5 | -1.25 | 1.11E-02 |
| 363 | ENSMUSG00000069515 | *LYZ1* | 980.75 | 529.25 | -1.24 | 5.46E-03 |
| 364 | ENSMUSG00000049112 | *OXTR* | 658.25 | 296.5 | -1.24 | 4.16E-03 |
| 365 | ENSMUSG00000033970 | *RFC3* | 205.5 | 98 | -1.24 | 8.40E-05 |
| 366 | ENSMUSG00000002870 | *MCM2* | 244.75 | 122 | -1.23 | 1.46E-03 |
| 367 | ENSMUSG00000052396 | *MOGAT2* | 108.25 | 50.5 | -1.23 | 1.14E-02 |
| 368 | ENSMUSG00000055541 | *Lair1* | 90.75 | 48.5 | -1.23 | 1.61E-02 |
| 369 | ENSMUSG00000035208 | *SLFN8* | 577.25 | 296.25 | -1.23 | 9.50E-04 |
| 370 | ENSMUSG00000026447 | *PIK3C2B* | 447.5 | 221.5 | -1.23 | 5.55E-03 |
| 371 | ENSMUSG00000036412 | *Arsi* | 444.5 | 248 | -1.22 | 7.44E-04 |
| 372 | ENSMUSG00000055745 | *LDOC1L* | 155.5 | 76 | -1.22 | 1.62E-04 |
| 373 | ENSMUSG00000053835 | *H2-T24* | 403.75 | 213 | -1.22 | 1.21E-02 |
| 374 | ENSMUSG00000035397 | *KLF16* | 450.5 | 227.75 | -1.22 | 5.18E-05 |
| 375 | ENSMUSG00000037035 | *INHBB* | 1088.5 | 500.75 | -1.21 | 3.54E-04 |
| 376 | ENSMUSG00000000317 | *BCL6B* | 1064 | 450.5 | -1.21 | 1.25E-03 |
| 377 | ENSMUSG00000031661 | *NKD1* | 260.25 | 129.75 | -1.21 | 2.79E-03 |
| 378 | ENSMUSG00000025854 | *FAM20C* | 675.75 | 300.5 | -1.21 | 5.38E-04 |
| 379 | ENSMUSG00000040287 | *STAC3* | 127.75 | 69.25 | -1.20 | 4.22E-02 |
| 380 | ENSMUSG00000020917 | *ACLY* | 106918 | 50867.25 | -1.20 | 2.29E-04 |
| 381 | ENSMUSG00000031934 | *Panx1* | 120.5 | 65 | -1.19 | 2.00E-02 |
| 382 | ENSMUSG00000002257 | *DEF6* | 296.75 | 162 | -1.19 | 1.46E-02 |
| 383 | ENSMUSG00000028480 | *Glipr2* | 536.5 | 291 | -1.18 | 8.13E-04 |
| 384 | ENSMUSG00000045667 | *SMTNL2* | 245 | 133 | -1.18 | 4.32E-02 |
| 385 | ENSMUSG00000030691 | *FCHSD2* | 777.5 | 414.5 | -1.18 | 7.39E-04 |
| 386 | ENSMUSG00000025352 | *Gdf11* | 144.25 | 81.25 | -1.18 | 2.60E-02 |
| 387 | ENSMUSG00000044667 | *PLPPR4* | 99.5 | 50.75 | -1.17 | 4.91E-02 |
| 388 | ENSMUSG00000047139 | *CD24A* | 436.25 | 238.75 | -1.17 | 1.79E-02 |
| 389 | ENSMUSG00000052125 | *F730043M19RIK* | 124 | 60.25 | -1.17 | 3.02E-03 |
| 390 | ENSMUSG00000074227 | *SPINT2* | 258.75 | 135 | -1.17 | 1.16E-02 |
| 391 | ENSMUSG00000051627 | *Hist1h1e* | 131.75 | 66 | -1.17 | 1.00E-02 |
| 392 | ENSMUSG00000024013 | *FGD2* | 603.25 | 337.75 | -1.17 | 7.38E-03 |
| 393 | ENSMUSG00000043872 | *ZMYM1* | 124 | 63.25 | -1.17 | 1.60E-04 |
| 394 | ENSMUSG00000039157 | *FAM102A* | 939.25 | 487 | -1.17 | 5.27E-04 |
| 395 | ENSMUSG00000039232 | *STX11* | 110 | 60.25 | -1.16 | 1.96E-02 |
| 396 | ENSMUSG00000024063 | *LBH* | 1887.25 | 983.75 | -1.16 | 7.88E-04 |
| 397 | ENSMUSG00000049313 | *Sorl1* | 5232.25 | 2585.5 | -1.16 | 1.92E-04 |
| 398 | ENSMUSG00000020941 | *MAP3K14* | 437.25 | 243.5 | -1.16 | 2.71E-03 |
| 399 | ENSMUSG00000037321 | *TAP1* | 2229.5 | 1175.5 | -1.16 | 1.10E-04 |
| 400 | ENSMUSG00000035692 | *Isg15* | 348.75 | 169 | -1.16 | 5.94E-04 |
| 401 | ENSMUSG00000031489 | *ADRB3* | 14768.75 | 7655 | -1.16 | 4.29E-08 |
| 402 | ENSMUSG00000024986 | *Hhex* | 214 | 113 | -1.15 | 2.21E-02 |
| 403 | ENSMUSG00000087107 | *AI662270* | 119 | 65.25 | -1.15 | 2.41E-02 |
| 404 | ENSMUSG00000055805 | *FMNL1* | 1244.5 | 685.25 | -1.15 | 2.18E-02 |
| 405 | ENSMUSG00000049744 | *ARHGAP15* | 162.25 | 93.25 | -1.15 | 4.31E-02 |
| 406 | ENSMUSG00000022436 | *SH3BP1* | 631.25 | 368 | -1.15 | 1.36E-02 |
| 407 | ENSMUSG00000026443 | *LRRN2* | 104 | 54.5 | -1.14 | 3.62E-03 |
| 408 | ENSMUSG00000045382 | *CXCR4* | 252.5 | 135.25 | -1.14 | 1.67E-02 |
| 409 | ENSMUSG00000028581 | *Laptm5* | 3563.75 | 2111 | -1.14 | 1.25E-02 |
| 410 | ENSMUSG00000037126 | *Psd* | 220.25 | 119.5 | -1.14 | 5.03E-03 |
| 411 | ENSMUSG00000022488 | *NCKAP1L* | 1109.75 | 648 | -1.14 | 1.36E-02 |
| 412 | ENSMUSG00000071042 | *RASGRP3* | 934.75 | 475.75 | -1.13 | 1.03E-04 |
| 413 | ENSMUSG00000022148 | *FYB* | 341 | 201.75 | -1.13 | 3.41E-02 |
| 414 | ENSMUSG00000036246 | *GMIP* | 927 | 518 | -1.13 | 7.01E-03 |
| 415 | ENSMUSG00000024300 | *MYO1F* | 431.5 | 256.75 | -1.13 | 3.14E-03 |
| 416 | ENSMUSG00000024691 | *Fam111a* | 746 | 413.25 | -1.13 | 3.49E-03 |
| 417 | ENSMUSG00000017692 | *RHBDL3* | 268 | 133 | -1.12 | 4.08E-03 |
| 418 | ENSMUSG00000038524 | *FCHSD1* | 140 | 79.75 | -1.11 | 1.59E-03 |
| 419 | ENSMUSG00000004814 | *CCL24* | 227.25 | 134 | -1.10 | 1.16E-02 |
| 420 | ENSMUSG00000053137 | *Mapk11* | 315.75 | 172.5 | -1.10 | 1.86E-02 |
| 421 | ENSMUSG00000075289 | *CARNS1* | 311.75 | 171.25 | -1.10 | 4.01E-03 |
| 422 | ENSMUSG00000020916 | *KRT36* | 117.75 | 63.25 | -1.10 | 2.69E-03 |
| 423 | ENSMUSG00000002228 | *PPM1J* | 97 | 50.25 | -1.10 | 8.57E-03 |
| 424 | ENSMUSG00000032020 | *UBASH3B* | 128.75 | 76 | -1.10 | 2.51E-02 |
| 425 | ENSMUSG00000031494 | *CD209A* | 206 | 118.25 | -1.09 | 1.05E-03 |
| 426 | ENSMUSG00000041920 | *Slc16a6* | 282.5 | 160.75 | -1.09 | 4.32E-03 |
| 427 | ENSMUSG00000037995 | *IGSF9* | 121.75 | 65 | -1.09 | 2.83E-03 |
| 428 | ENSMUSG00000032436 | *CMTM7* | 289.25 | 171.25 | -1.09 | 6.85E-03 |
| 429 | ENSMUSG00000024610 | *Cd74* | 17194.5 | 10194.5 | -1.09 | 1.06E-02 |
| 430 | ENSMUSG00000025402 | *NAB2* | 301 | 162.25 | -1.09 | 8.86E-04 |
| 431 | ENSMUSG00000037649 | *H2-DMA* | 610 | 356.75 | -1.09 | 1.23E-02 |
| 432 | ENSMUSG00000030789 | *ITGAX* | 188.5 | 114.5 | -1.09 | 2.20E-02 |
| 433 | ENSMUSG00000050379 | *SEPT6* | 657.75 | 360.75 | -1.09 | 7.98E-03 |
| 434 | ENSMUSG00000002058 | *UNC119* | 880.5 | 444.75 | -1.08 | 2.89E-04 |
| 435 | ENSMUSG00000097730 | *GM26588* | 210.75 | 108.25 | -1.08 | 2.48E-03 |
| 436 | ENSMUSG00000018819 | *Lsp1* | 2391.5 | 1446.25 | -1.08 | 6.74E-03 |
| 437 | ENSMUSG00000024397 | *Aif1* | 170.75 | 101.75 | -1.08 | 1.51E-02 |
| 438 | ENSMUSG00000005410 | *MCM5* | 220.25 | 122.5 | -1.07 | 1.46E-02 |
| 439 | ENSMUSG00000049625 | *Tifab* | 162.75 | 97.25 | -1.07 | 7.53E-03 |
| 440 | ENSMUSG00000028976 | *SLC2A5* | 1335.25 | 642.25 | -1.06 | 1.40E-02 |
| 441 | ENSMUSG00000032812 | *Arap1* | 1602 | 937 | -1.06 | 1.36E-04 |
| 442 | ENSMUSG00000066154 | *Mup3* | 171.5 | 99 | -1.06 | 7.51E-03 |
| 443 | ENSMUSG00000039126 | *PRUNE2* | 788.75 | 402.5 | -1.06 | 1.57E-03 |
| 444 | ENSMUSG00000027797 | *DCLK1* | 380 | 217.5 | -1.06 | 1.66E-03 |
| 445 | ENSMUSG00000070034 | *SP110* | 962 | 566 | -1.06 | 1.74E-02 |
| 446 | ENSMUSG00000047767 | *Atg16l2* | 304.5 | 173.5 | -1.06 | 1.14E-02 |
| 447 | ENSMUSG00000002083 | *BBC3* | 489 | 279 | -1.06 | 8.71E-04 |
| 448 | ENSMUSG00000032915 | *ADGRE4* | 81 | 44.75 | -1.05 | 7.82E-03 |
| 449 | ENSMUSG00000047182 | *IRS3* | 1456.75 | 828.75 | -1.05 | 1.15E-04 |
| 450 | ENSMUSG00000015340 | *CYBB* | 844.5 | 527.5 | -1.05 | 8.90E-03 |
| 451 | ENSMUSG00000004947 | *DTX2* | 2283.25 | 1283.25 | -1.05 | 2.11E-06 |
| 452 | ENSMUSG00000060791 | *GMFG* | 361.5 | 217.5 | -1.05 | 2.31E-02 |
| 453 | ENSMUSG00000079547 | *H2-DMB1* | 653.5 | 395.5 | -1.05 | 1.20E-02 |
| 454 | ENSMUSG00000031790 | *Mmp15* | 1198.5 | 655.5 | -1.05 | 8.37E-05 |
| 455 | ENSMUSG00000058454 | *DHCR7* | 959 | 500.75 | -1.05 | 5.16E-04 |
| 456 | ENSMUSG00000021423 | *LY86* | 350.25 | 222.25 | -1.04 | 2.13E-02 |
| 457 | ENSMUSG00000040616 | *TMEM51* | 102.75 | 60 | -1.04 | 1.57E-02 |
| 458 | ENSMUSG00000072620 | *SLFN2* | 863 | 526.5 | -1.04 | 2.54E-02 |
| 459 | ENSMUSG00000091649 | *PHF11B* | 183.5 | 108.5 | -1.04 | 2.21E-02 |
| 460 | ENSMUSG00000058099 | *Nfam1* | 362.25 | 231.25 | -1.04 | 1.09E-02 |
| 461 | ENSMUSG00000004609 | *Cd33* | 402.25 | 246.75 | -1.03 | 7.44E-03 |
| 462 | ENSMUSG00000021594 | *SRD5A1* | 99 | 61.75 | -1.03 | 6.17E-03 |
| 463 | ENSMUSG00000020689 | *ITGB3* | 272 | 164.25 | -1.03 | 9.40E-03 |
| 464 | ENSMUSG00000045312 | *LHFPL2* | 2803.25 | 1580 | -1.03 | 3.87E-04 |
| 465 | ENSMUSG00000031434 | *MORC4* | 1059.25 | 579.25 | -1.02 | 4.37E-04 |
| 466 | ENSMUSG00000004098 | *COL5A3* | 25584.5 | 13960.75 | -1.02 | 2.21E-04 |
| 467 | ENSMUSG00000028159 | *DAPP1* | 207.25 | 125 | -1.02 | 2.56E-02 |
| 468 | ENSMUSG00000041849 | *Card6* | 355.5 | 213.25 | -1.02 | 1.82E-02 |
| 469 | ENSMUSG00000013707 | *TNFAIP8L2* | 202.5 | 134 | -1.02 | 3.79E-02 |
| 470 | ENSMUSG00000009772 | *NUAK2* | 184 | 116 | -1.02 | 3.46E-02 |
| 471 | ENSMUSG00000032688 | *MALT1* | 855 | 486.25 | -1.02 | 5.27E-03 |
| 472 | ENSMUSG00000087500 | *Gm12426* | 76.5 | 39 | -1.01 | 2.06E-02 |
| 473 | ENSMUSG00000038807 | *RAP1GAP2* | 570.25 | 303.5 | -1.01 | 1.41E-03 |
| 474 | ENSMUSG00000021457 | *SYK* | 1649.5 | 1055 | -1.01 | 2.88E-02 |
| 475 | ENSMUSG00000034758 | *TLE6* | 71.75 | 41.75 | -1.01 | 3.19E-03 |
| 476 | ENSMUSG00000097187 | *GM19426* | 86 | 48 | -1.01 | 1.10E-02 |
| 477 | ENSMUSG00000001604 | *Tcea3* | 132.75 | 81 | -1.01 | 4.70E-02 |
| 478 | ENSMUSG00000115100 | *Gm34934* | 164 | 86.75 | -1.01 | 5.47E-03 |
| 479 | ENSMUSG00000069892 | *9930111J21RIK2* | 488.25 | 292.75 | -1.01 | 3.54E-02 |
| 480 | ENSMUSG00000022203 | *EFS* | 908.5 | 542 | -1.01 | 4.16E-04 |
| 481 | ENSMUSG00000019856 | *FAM184A* | 105.5 | 57.75 | -1.01 | 1.09E-02 |
| 482 | ENSMUSG00000006403 | *ADAMTS4* | 190.25 | 100.25 | -1.01 | 2.53E-02 |
| 483 | ENSMUSG00000031659 | *ADCY7* | 1270.25 | 804 | -1.01 | 1.17E-02 |
| 484 | ENSMUSG00000024613 | *TCOF1* | 885.25 | 532.5 | -1.01 | 2.58E-03 |
| 485 | ENSMUSG00000054871 | *TMEM158* | 112.25 | 67.75 | -1.01 | 6.96E-03 |
| 486 | ENSMUSG00000054435 | *GIMAP4* | 1308.75 | 747.5 | -1.01 | 2.77E-02 |
| 487 | ENSMUSG00000024349 | *TMEM173* | 553.75 | 340.75 | -1.00 | 6.28E-03 |
| 488 | ENSMUSG00000031827 | *COTL1* | 1422.25 | 912.5 | -1.00 | 1.07E-02 |
| 489 | ENSMUSG00000000838 | *FMR1* | 4932.25 | 2654.5 | -1.00 | 4.81E-04 |
| 490 | ENSMUSG00000030844 | *Rgs10* | 331.25 | 211.5 | -1.00 | 1.15E-02 |
| 491 | ENSMUSG00000067370 | *B3GALT4* | 69.5 | 42.25 | -1.00 | 2.39E-02 |
| 492 | ENSMUSG00000005470 | *ASF1B* | 80.75 | 51.25 | -1.00 | 1.97E-02 |
| 493 | ENSMUSG00000067586 | *S1PR3* | 507.75 | 296.5 | -1.00 | 8.44E-04 |
